# Hidden diversity in forest soils: Characterization and comparison of terrestrial flatworm’s communities in two national parks in Spain

**DOI:** 10.1002/ece3.4178

**Published:** 2018-07-02

**Authors:** Marta Álvarez‐Presas, Eduardo Mateos, Marta Riutort

**Affiliations:** ^1^ Departament de Genètica, Microbiologia i Estadística Institut de Recerca de la Biodiversitat (IRBio) Universitat de Barcelona Barcelona Spain; ^2^ Departament de Biologia Evolutiva, Ecologia i Ciències Ambientals Universitat de Barcelona Barcelona Spain

**Keywords:** Last Glacial Maximum, *Microplana*, molecular phylogenetics, refugia, soil ecology, species diversity

## Abstract

Terrestrial flatworms (Platyhelminthes, Tricladida, and Geoplanidae) belong to what is known as cryptic soil fauna of humid forests and are animals not easily found or captured in traps. Nonetheless, they have been demonstrated to be good indicators of the conservation status of their habitat as well as a good model to reconstruct the recent and old events affecting biodiversity. This is mainly due to their delicate constitution, their dependence on the integrity of their habitat, and their very low dispersal capacity. At present, little is known about their communities, except for some studies performed in Brazil. In this work, we analyze for the first time in Europe terrestrial flatworm communities. We have selected two protected areas belonging to the *Red Española de Parques Nacionales*. Our aims include performing a first study of the species richness and community structure for European terrestrial planarian species at regional and local scale. We evaluate the effect of type of forests in the community composition and flatworms’ abundance, but also have into account the phylogenetic framework (never considered in previous studies) analyzed based on molecular data. We find differences in the species composition among parks, with an astonishingly high diversity of endemic species in the *Parque Nacional de Picos de Europa* and an extremely low diversity of species in the *Parque Nacional de Ordesa y Monte Perdido*. These divergent patterns cannot be attributed to differences in physical variables, and in addition, the analyses of their phylogenetic relationships and, for a few species, their genetic structure, point to a more probable historical explanation.

## 1 INTRODUCTION

Soil fauna communities generally present a structure that is caused by different factors depending on the spatial scales (Ettema & Wardle, 2002). Principal biological factors are types of vegetation, food resources availability, and interactions of animal species with other organisms, especially microorganisms (Lavelle & Spain, 2001). Abiotic factors, such as bedrock composition, microsite humidity, mean annual precipitation, or type of forest cover, can result in variations of abundance and species composition in the soils at different scales, from microsite, site, local to regional levels (Melguizo‐Ruiz, Verdeny‐Vilalta, Arnedo, & Moya‐Laraño, 2012). On the other hand, taxa from soil communities can exhibit also the genetic imprint of ancient climatic and geographic events that may have been lost in other organisms with higher dispersal capacity (Pfenninger & Posada, 2002; Sunnucks et al., 2006). As a consequence, these groups of organisms allow the reconstruction of old events affecting the generation and maintenance of biodiversity and become excellent indicators of the conservation status of forest soils. However, to be used for such aim, an extensive knowledge about the state and functioning of their communities is necessary. Land planarians (Platyhelminthes, Tricladida, Geoplanidae) belong to this group of animals; they are inhabitants of humid forests soils and top predators of other invertebrates. They are simple animals that do not possess mechanisms for water retention; therefore, they are dependent on soil moisture to maintain their water requirements and use vertical migration through soil, litter, and vegetation to keep their humidity (Winsor, Johns, & Yeates, 1998). Land planarians are in general sensible to disturbed habitats, although some are reported to be adapted to inhabit them (Carbayo, Leal‐Zanchet, & Vieira, 2002; Oliveira et al., 2014; Álvarez‐Presas, Amaral, Carbayo, Leal‐Zanchet, & Riutort, 2015). Based on these features, some studies have highlighted the value of this group of organisms as bioindicators in relation to the habitat perturbations caused by human activities (Sluys, 1998).

The highest species richness of autochthonous land flatworms worldwide has been documented in the southern hemisphere (Winsor et al., 1998), especially in areas originally covered by the south‐eastern Brazilian Atlantic Rain Forest (Carbayo et al., 2002; Fick, Leal‐Zanchet, & Vieira, 2006; Fonseca et al., 2009; Leal‐Zanchet & Baptista, 2009; Sluys, 1998, 1999). This could, of course, be in part related to the existence of research teams interested in the group. Probably due to this bias also most studies on communities for this group have been performed in a restricted number of very specific areas in South America: ombrophilous forests, deciduous, and semideciduous forests in Southern Brazil (Antunes, 2008; Baptista, de Matos, Fick, & Leal‐Zanchet, 2006; Baptista & Leal‐Zanchet, 2010; Carbayo, Leal‐Zanchet, & Vieira, 2001; Carbayo et al., 2002; De Castro & Leal‐zanchet, 2005; Fick et al., 2006; Leal‐Zanchet & Baptista, 2009; Leal‐Zanchet, Baptista, Campos, & Raffo, 2011; Leal‐Zanchet & Carbayo, 2000, 2001; Palacios, Baptista, & Leal‐Zanchet, 2006) and in the Atlantic Forest of northern Argentina (Negrete, Colpo, & Brusa, 2014). Other studies have analyzed the ecology of introduced terrestrial planarian communities in Europe in relation to their invasive capacity (Boag, Yeates, & Johns, 1998; Boag, Jones et al., 1998; Christensen & Mather, 1998; Jones, Green, & Palin, 1998; Yeates, Boag, & Johns, 1997), but no study on community composition or ecology of autochthonous European terrestrial planarians has been performed.

Studies conducted in Brazil have shown that the community structure for terrestrial flatworms can be influenced by the vegetation type and by the degree of anthropic alteration (Carbayo et al., 2002; Fick et al., 2006; Fonseca et al., 2009). However, studies analyzing the effect of environmental factors have not found any of them as driver of the abundance or species composition of terrestrial planarians communities (Antunes, Leal‐Zanchet, & Fonseca, 2012; Baptista & Leal‐Zanchet, 2010; Boag, Jones et al., 1998; Boag, Yeated et al., 1998; Fick et al., 2006; Johns, Boag, & Yeates, 1998; Sluys, 1998; Winsor et al., 1998) with the only exception of pH and organic matter (see Baptista & Leal‐Zanchet, 2010). At last, none of the community studies conducted so far has taken into account the phylogenetic relationships between the species found, nor whether these relationships or the climatic and geological history of the area can explain communities’ composition differences among areas. Moreover, none has used molecular data in conjunction with phylogenetic inference methods to delineate genetic lineages, which also provides a more accurate delimitation of molecular operational taxonomic units (MOTUs) or species and avoids the problem of identifying morphologically cryptic or pseudocryptic species (common in terrestrial planarians, Álvarez‐Presas et al., 2015; Carbayo, Álvarez‐Presas, Jones, & Riutort, 2016; Amaral et al., 2018) that may take much time and lengthen the process of community study.

In Europe, although the diversity of species is still much lower than in tropical regions, recent publications have shown that the species richness of this group in this temperate region is higher than previously suspected (Mateos, Sluys, Riutort, & Álvarez‐Presas, 2017; Sluys, Mateos, Riutort, & Álvarez‐Presas, 2016; and references therein). In this continent, all the native species belong to the subfamily Microplaninae and to a single genus, *Microplana;* although there are some doubts about whether the genus *Rhyncodemus* can also be autochthonous (Jones, 1988, 1998; Ogren & Kawakatsu, 1998). The European species are in general much smaller than the tropical ones and less colorful, which renders them less prone to be found and identified in any soil community study. Moreover, the fact that this group of animals belong to cryptic soil fauna, due to the difficulty in sampling them by the usual methodologies as using traps or soil fauna extractors, makes difficult their inclusion in any study of communities, nonetheless, they can contribute important information.

In Spain, the forests suitable for terrestrial planarians are few and are located mainly in the north of the Peninsula. In that region, three national parks bear forests with the characteristics needed to host terrestrial planarians: in the Pyrenees, the national parks *Aigüestortes i Sant Maurici* and *Ordesa y Monte Perdido*, and in the Cantabrian Mountains *Picos de Europa*. The two latter present a broader extension of these types of forests and are where we have focused our study. The *Red Española de Parques Nacionales* is an integrated system intended to protect and manage a selection of the best samples of the Spanish Natural Patrimony (http://www.mapama.gob.es/es/red-parques-nacionales/ last visited January 2018). Among its objectives, one of the most important is the protection and management of its biodiversity in order to ensure the proper functioning of ecosystems. It is desirable therefore that they house a biological diversity that is representative of its original biodiversity (Araújo, Lobo, & Moreno, 2007), that includes the levels of genetic diversity needed for the maintenance of its populations and that it is also representative of the diversity in the region. But for this it is necessary to know what was the original situation of the fauna inhabiting them, knowledge that is currently inexistent for the terrestrial planarian communities, as well as for other cryptic forest soil dwellers.

In this work, we performed a study on the diversity of terrestrial planarian communities focusing on two national parks of the *Red Española de Parques Nacionales*:* Ordesa y Monte Perdido* (hereafter referred as Ordesa) and *Picos de Europa* (hereafter referred as Picos). These parks have been selected because they are located in the area of the Iberian Peninsula with the highest probability of housing terrestrial planarians, as explained above. The broader extent of Picos and its higher diversity of forests may influence the genetic diversity distribution between and within the parks, predicting finding a higher diversity and species richness in Picos. In addition, the two parks are situated in regions that have gone through different ancient climatic events (Hewitt, 1999; Petit, Brewer, Bordács, & Burg, 2002; Petit et al., 2003) also pointing to an expectation of a higher genetic diversity in Picos than in Ordesa, which could be reflected in a higher number of species and/or within species diversity. Thus, the specific aims of the study are as follows: (i) performing a first analysis of the species richness and the community structure for European terrestrial planarian species at a regional scale; (ii) analyze the effect of the forest type in their communities (local scale); and (iii) explore the drivers of the communities composition in the parks under a phylogenetic framework.

## 2 MATERIALS AND METHODS

### 2.1 Study area

**Figure 1:**
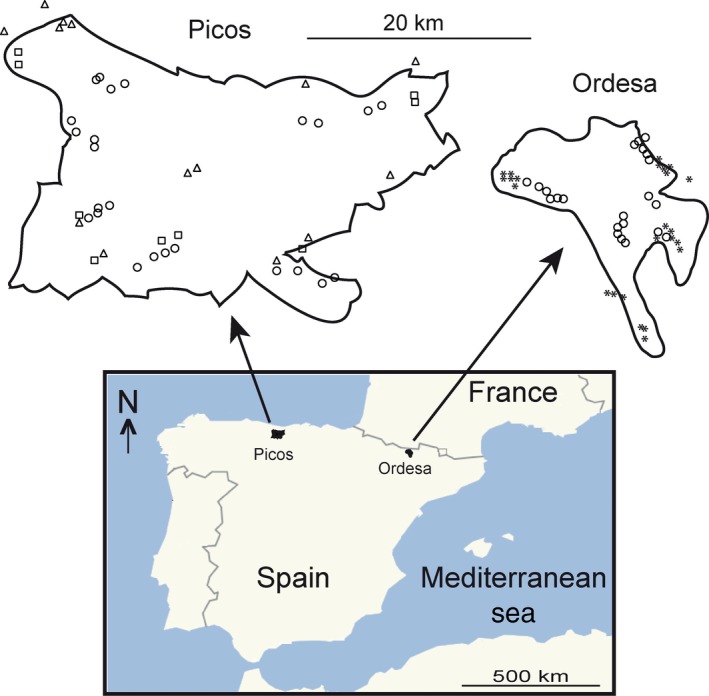
Map showing the situation within Spain of the two National Parks, and a detail of the distribution of plots within each park. Picos and Ordesa map at the same scale. Squares, oak forest; triangles, mixed forest; circles, beech forest; asterisks, pine forest

The study area comprises two national parks in Northern Spain (Figure 1): *Ordesa y Monte Perdido* (42°40′N, 0°3′E) and *Picos de Europa* (43°30′N, 4°55′W). Ordesa, within the Aragonese Pyrenees, in the Huesca province, extends across 15,636 ha and exhibits a mixture of climates, with both Mediterranean and Oceanic influences, and with a mean annual rainfall range between 1,129 and 1,690 mm/year. The highlands of the park (above 2,000 m altitude) are extremely arid, as all water from rainfall is quickly picked up by the karstic system. On the contrary, valley bottoms are covered with lush vegetation (forested area occupies 21% of the park range) dominated by beech (*Fagus sylvatica*, 7.8%), pine forest of *Pinus sylvestris* (6.6%), and fir trees giving way to the mountain pine (*Pinus uncinata*) as altitude increases (Benito Alonso, 2010). Our sites were located at elevations from 991 to 1618 m a.s.l. and equally spread across the four main valleys composing the national park (Añisclo, Escuaín, Ordesa, and Pineta valleys).

Picos extends across Asturias, León, and Cantabria provinces, in the Cantabrian mountain range. It has a surface area of 67,127 ha, with a pronounced influence of Oceanic climate (Atlantic climate), with cool summers and comparatively warm winters (Felicísimo, 1994). It has the highest limestone formation in Atlantic Europe, with important karstic processes, chasms reaching more than 1,000 m, very clear glacial erosion and presence of lakes. It is characterized by a narrower range of annual temperatures than those encountered in Ordesa at comparable latitudes, lacking, for instance, the extremely dry summers typical of the other park, which is more influenced by Mediterranean climate, and with a mean annual precipitation between 1,109 and 1,968 mm/year. Unlike Ordesa, most rainfall on Picos comes as drizzle, and mountain fogs are very frequent due to the Oceanic influence (Felicísimo, 1994). Forested area occupies 25% of the park’s range, being beech forests of *Fagus sylvatica* the most abundant forest type (18.4% of the park area), followed by mixed and oak forests (<0.5%). Atlantic mixed forests of Picos, relics difficult to find in Spain, appear on the lower part of the mountain and are interspersed with meadows areas. Common and cornish oaks (*Quercus robur* or *Quercus petraea*) and hazels (*Corylus avellana*) are intermingled with birch (*Betula celtiberica*) as the main tree species (5%), maples (*Acer* sp.), linden (*Tillia* sp.), ash (*Fraxinus excelsior)*, chestnut (*Castanea* sp.), and walnut (*Juglans regia*) trees; at its feet, an undergrowth of brambles, briars, and thorn. Our collection sites were established at elevations ranging from 115 to 1,353 m a.s.l. distributed in the three main areas composing the park (western, central, and eastern massifs).

### 2.2 Collection sites and sampling protocol

When working with cryptic soil fauna, one important question is the establishment of a standard sampling unit and the sampling methodology used in order to get comparable data between these sampling units. The authors working on terrestrial flatworm’s ecology (see Baptista & Leal‐Zanchet, 2010; Baptista et al., 2006; Carbayo et al., 2002; De Castro & Leal‐zanchet, 2005; Fick et al., 2006; Leal‐Zanchet et al., 2011) used plots of fixed sizes (50 × 2 m, 2 × 2 m, 7 × 7 m) or transects of fixed length (30 to 50 m) as a sampling unit. On each plot or transect, the sampling methodology used was always direct searching by a fixed number of researchers (between one and five) during a standardized lapse of time (between 15 min and 1 hr). In our study, each sampling unit consisted of a forest plot (of approximately 50 × 50 m) where two people searched for terrestrial flatworms actively during one hour under logs and stones. Each planarian detected was captured alive and deposited in a little plastic container with humid substrate.

At first, we stablished in 24 the number of plots to be sampled on each forest type of each national park. However, it was not always possible to sample in the 24 plots selected a priori. In all cases, the selection of the plots had into account a visual inspection of the humidity of the site, selecting always the wetter sites maximizing the probability of encountering terrestrial planarians. In Picos (Figure 1), three forest types were selected as follows: beech forest (24 plots), oak forest (nine plots, main tree species *Quercus robur* in two plots, and *Quercus petraea* in seven plots), and mixed forest (14 plots, main tree species *Quercus robur* mixed with *Fraxinus excelsior* and *Betula celtiberica* in five plots, and *Quercus petraea* mixed with *Fraxinus excelsior* and *Betula celtiberica* in nine plots). In Picos, only nine plots of oak forests and 14 plots of mixed forest were sampled because of the limited availability of forests patches that met the appropriate conditions for sampling. In Ordesa (Figure 1), samplings were performed in two forest types: beech forest (22 plots) and pine forest, (24 plots). In this park, two plots of beech forest were not sampled due to the deterioration status of the forest patch selected. All sampling protocol was performed twice (dates in format day/month/year), from 2/10/2013 to 13/10/2013 and from 24/5/2014 to 4/6/2014 in Ordesa, and from 21/10/2013 to 01/11/2013 and from 22/6/2014 to 03/7/2014 in Picos. Every sampling day, plots of different forests type were sampled in order to avoid temporary self‐replication. The data of the two sampling campaigns were pooled.

In the same day of the collections, the animals were visualized under a stereomicroscope, their morphological external appearance recorded and photographed, and finally fixed. When the specimens were big enough, a small anterior section was fixed in absolute ethanol for DNA extraction, and the rest (including the parts necessary for histological studies) were fixed with Steinmann fluid and stored in 70% alcohol in order to study the copulatory apparatus and other structures that allow the diagnosis of the species.

### 2.3 Environmental parameters

Pluviometry data were obtained from two meteorological stations located at the east and west ends of each national park. Total annual rainfall as well as accumulated rainfall of the 3 months previous to each sampling campaign was recorded. For calculation purposes, the mean value of the two stations of each park was considered. In Ordesa, the two meteorological stations were located in Bielsa municipality (at 1,100 m asl) and Torla municipality (1,000 m asl). In Picos, the two meteorological stations were located in Soto de Sajambre municipality (1,500 m asl) and Sotres municipality (1,200 m asl). On each sampling campaign soil temperature, pH and water content were measured. On each plot, soil temperature was taken in three points and the mean value was used for calculations. Also on each plot, two samples of soil substrate were taken to measure hydric content and two other samples were taken to measure the pH in the laboratory; the mean values of each variable were used for calculations. Measures of pH were performed the same day with a portable pH meter (PH25, Crison) by diluting the soil substrate samples in 5 volumes of distilled water. Also the same day, the two soil samples for hydric content were weighted to obtain their fresh weight (Fw). Once back in the University laboratory, the samples were dried in a stove at 105°C for 48 hr and then were weighted again to have their dry weight (Dw). The hydric content for each sample was expressed in percentage of water content and calculated gravimetrically by the equation 100*[(Fw‐Dw)/Fw].

### 2.4 Species assignation and phylogenetic inference

Samples preserved in 100% ethanol were used for DNA extraction with the Wizard^®^ Genomic purification kit (Promega, Madison, WI, USA) following the same protocol as in Álvarez‐Presas, Carbayo, Rozas, and Riutort (2011). A fragment of the gene encoding the mitochondrial cytochrome oxidase I (Cox1) was analyzed by polymerase chain reaction (PCR) together with three nuclear genes: genes encoding the 18S type II rRNA (18S) and 28S rRNA (28S) and a fragment of Elongation Factor 1‐alpha (EF). For 18S and 28S, we used primers and PCR conditions as in Álvarez‐Presas, Baguñà, and Riutort (2008) for Cox1 as in Álvarez‐Presas et al. (2011) and for the EF as in Carbayo et al. (2013). The same primers were used for PCR amplification and sequencing. The amplification products were purified directly with a vacuum pump (Multiscreen^®^HTS Vacuum Manifold, Millipore Corporation, Billerica, MA 01821, USA). DNA sequences were determined from both strands by Sanger sequencing in Macrogen (Amsterdam, Europe). Chromatograms were revised and contigs constructed in Geneious v 8.1.7. software (Biomatters; available from http://www.geneious.com last visited January 2018).

Genes Cox1 and EF were aligned based on the amino acid sequences using the Clustal W plugin included in the BioEdit software. 7.0.9.0. (Hall, 1999). Ribosomal RNA gene sequences were aligned using the online version of the software Mafft v. 7 (Katoh & Standley, 2013) applying the G‐INS‐i iterative refinement method and, subsequently, checked the alignments by eye with Bioedit. Positions that could not be unambiguously aligned were subsequently excluded from the analyses by applying GBlocks v 0.91b (Talavera & Castresana, 2007), with half allowed gap positions and a minimum length of a block of 10 nucleotides, to obtain the maximum number of nucleotides. Based on these alignments, we estimated the DNA sequence evolution model that better fits the data by using jModelTest v. 2.1.4. (Darriba, Taboada, Doallo, & Posada, 2012), applying the Akaike information criterion (AIC).

For the Cox1 gene, a saturation test was run using the software DAMBE6 (Xia, 2017) by plotting observed transitions and transversions vs. gene divergences under the GTR model.

Two datasets have been used for different analyses: (i) Cox1 dataset, included all Cox1 mitochondrial gene sequences to assign individuals from Picos and Ordesa to MOTUs; (ii) concatenated dataset, included the information of the three nuclear genes to infer a general phylogeny. Both alignments included sequences downloaded from GenBank (see Supplementary Information Table S1 for the individuals included in each dataset).

Phylogenies of the two datasets were inferred applying the maximum‐likelihood method (ML) with the software RaxML v.8 (Stamatakis, 2014) estimating bootstrap support values from 10,000 replicates. We also used the Bayesian inference method (BI) as implemented in the software MrBayes v3.2.2. (Ronquist et al., 2012) for the concatenated dataset. Two runs were applied producing 5 million generations for each and of these a tree each 1,000 was stored. A 25% default burn‐in was used, likelihood values (log‐likelihood) of cold chains were checked to have reached stationarity, and the convergence of the two runs was verified by the average standard deviation of split frequencies being ≪0.01. A consensus tree from the remaining trees was obtained. For the Cox1 dataset, we used BEAST software v2.3.2 (Bouckaert et al., 2014) since with MrBayes, it was impossible to reach the convergence of the two runs. A relaxed log normal clock was applied, and 100 million generations were run storing a tree of every 10,000.

For the MOTUS shared by the two parks and with a higher abundance (M02 and M78), haplotype networks were constructed using the median‐joining method (Bandelt, Forster, & Röhl, 1999) as implemented in the Popart program (available at http://popart.otago.ac.nz, last visited November 2017). To perform these haplotype networks, information from the Cox1 dataset was used, adding sequences from a previous analysis of *Microplana terrestris* in the Iberian peninsula (Álvarez‐Presas, Mateos, Vila‐Farré, Sluys, & Riutort, 2012) in the case of M02, and adding known sequences from *M. aixandrei* from the GenBank database, in the case of M78 (see Supporting information Table S1).

### 2.5 Numerical methods

On each plot, the measure unit in numerical analyses was the terrestrial flatworm abundance, defined as the number of specimens collected by two researchers during one hour in the two sampling campaigns pooled. We also quantified the species richness (number of species or MOTUS) per sampling plot. These two variables were compared among the parks and the forest types by ANOVA or *t*‐Student tests (with Tukey test post hoc comparisons when necessary) after checking the homogeneity of variances by the Levene test (Levene, 1960). A Pearson correlation test between soil water content, temperature, and pH with respect to flatworm abundance and richness per plot was performed. These tests were performed using R‐language package (Team, 2003).

From the matrix of abundance data of flatworm species (eliminating species with overall abundance inferior to three specimens), we carried out an analysis of similarity in flatworm species composition between national parks and among forest types. To do this analysis, the abundance data were pooled by forest type and transformed using log(*x* + 1) procedure. Differences in flatworm species composition between pairs of sampling points were quantified by the Bray–Curtis similarity index. From the similarity matrix, we performed a similarity analysis (ANOSIM, PRIMER‐E 2001), which gives a general *R*‐value and allows pairwise comparisons between the areas compared (parks or forest types).

For each national park, we estimated the three basic components of the diversity (sensu Whittaker, 1960). We define α‐diversity as the diversity measured on each forest type, β‐diversity as the species turnover between the forest types of each national park, and γ‐diversity as the diversity value measured on each national park as a whole (pooling the forest types). Estimates of α‐ and γ‐diversity were done using Shannon–Weiner diversity index (H), and species richness. β diversity was estimated using the Sørensen dissimilarity index between pairs of forests type on each national park; this index, defined as β_*t*_ in Wilson and Shmida (1984), is bound between 0 and 1, where 0 means that the two sites have the same composition (i.e., they share all the species), and 1 means that the two sites do not share any species. Commands “diversity” (with base 2 logarithms) and “betadiver,” from R Vegan package, were used for diversity calculations.

We have used species accumulation curves to model the species increase in relation to sample size at two different scale levels. First, analyzing the two national parks as a whole and second analyzing only beech forests of the two parks (as this is the shared forest type in the two parks). Due to the disparity in the sampled area (Picos is 4.3 times higher than Ordesa), in order to make comparable the species accumulation curves between beech forests in the two parks, the second analysis has been performed constructing four different plot data matrices in Picos, each of them with surface area equivalent to that found in Ordesa (see Supporting information Figure S1): north (12 plots, A + B), south (12 plots, C + D), east (eight plots, B + D), and west (16 plots, A + C). For the two species accumulation curves analyses we used the “method = random” from the command “specaccum” in R Vegan package, calculating the mean species accumulation curves and its standard deviation from random permutations of the sampling units (plots).

In general, in sampling protocols, not all species are detected in any site, and these unseen species also belong to the species pool (Oksanen, 2016). In order to estimate the number of unseen species on each forest type and on each national park as a whole, command “specpool” of R Vegan package was used. Command “specpool” studies a collection of sites and assumes that the number of unseen species is related to the number of rare species, or species seen only once or twice (see Oksanen, 2016 for a detailed discussion). Command “specpool” implements several nonparametric estimators of which we selected the Chao 1 estimator (Chao, 1987; Chiu, Wang, Walther, & Chao, 2014), the first order jackknife estimator, and the bootstrap estimator (Smith & van Belle, 1984), calculating the mean estimate values, and associate variances.

## 3 RESULTS

### 3.1 Species‐MOTUs

A total of 350 individuals belonging to the subfamily Microplaninae were collected from across all the sampling plots (Supporting information Tables S1 and S2), 202 from Ordesa and 148 from Picos.

**Figure 2:**
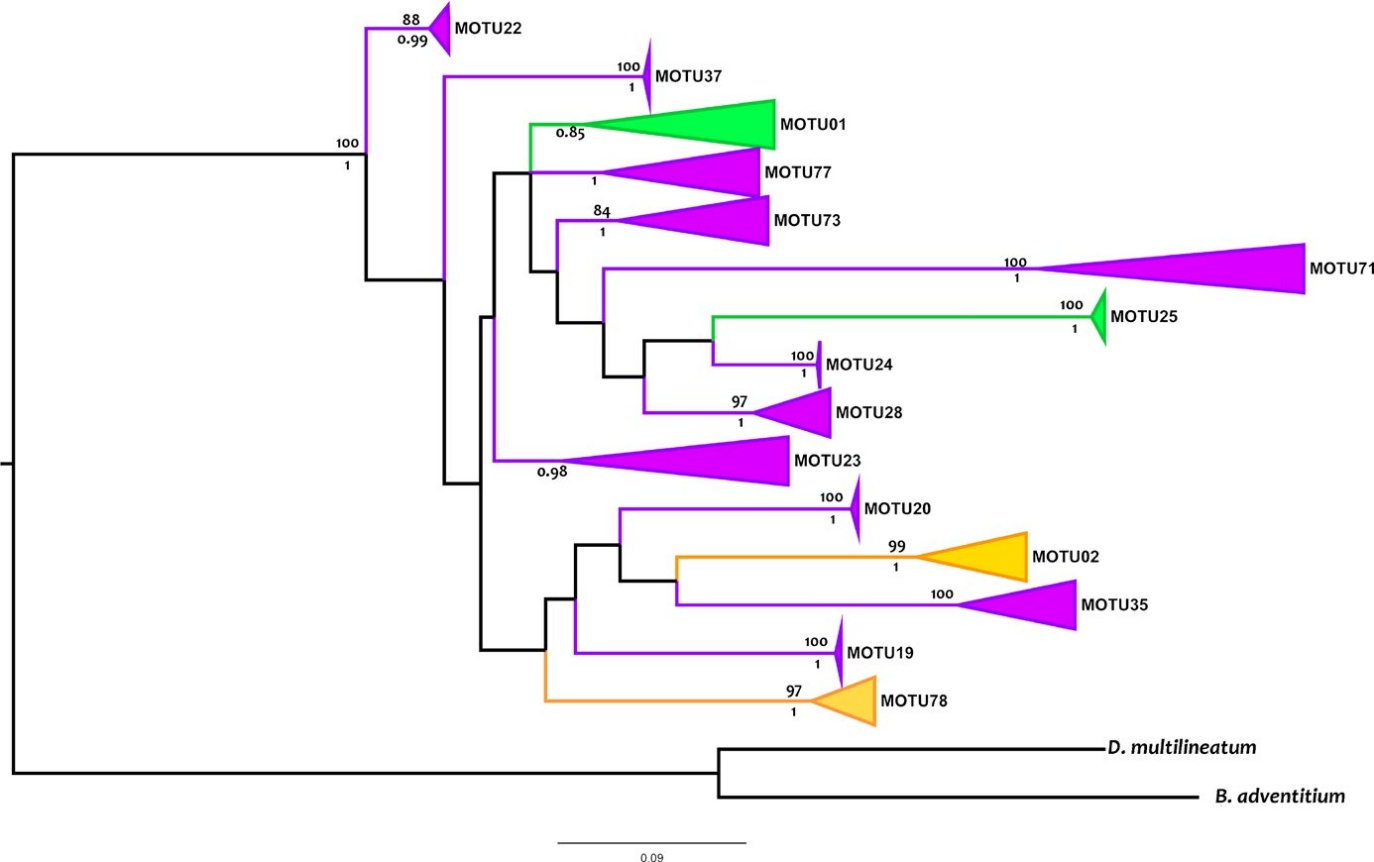
Maximum‐likelihood (ML) tree inferred from Cox1 dataset. Monophyletic groups comprising MOTUs have been collapsed. Values at nodes correspond to bootstrap values (above, ML analysis) and posterior probability (below, BI analysis); only values over 0.85 PP and 75% bootstrap are displayed, respectively. Scale bar = number of substitutions per site

The phylogenetic tree obtained with Cox1 gene sequences (Figure 2) defines 15 monophyletic groups or entities (MOTUs), four of them present in Ordesa and 13 in Picos (two are present in both parks). Five of the clades correspond to already described species, *M. terrestris* (M02), *M. nana* (M01), *M. fuscomaculosa* (M25), *M. nervosa* (M22), and *M*. cf. *aixandrei‐*2 (M78; Mateos et al., 2017). The rest here designated are under study and are going to be described as new species elsewhere (Álvarez‐Presas et al. *in preparation*). Nonetheless, in this work, we will designate all of them with their MOTU codes (Mxx).

Of the total of 15 MOTUs found, M02 (*M. terrestris*) and M78 (*M. *cf. *aixandrei*‐2) are the only two species shared in the two parks. In Ordesa, we have found four MOTUs all of them having a known distribution through Europe or wide in the Iberian Peninsula, not being in any case endemic from the Park or the area (Table 1). On the other hand, in Picos, seven of the 13 MOTUs found are endemic from the Park, three are found only in wet forests in North‐Western Spain, and three have a wide distribution through Europe (Table 1).

**Table 1:** Mean abundance of terrestrial flatworm MOTUs by forest type on each National Park

Park	Forest	*n*	*n*‐tp	M01	M02	M19	M20	M22	M23	M24	M25	M28	M35	M37	M71	M73	M77	M78
Ordesa	Beech	22	20	0.09	2.73	–	–	–	–	–	0.23	–	–	–	–	–	–	1.00
Ordesa	Pine	24	21	0.17	2.96	–	–	–	–	–	0.17	–	–	–	–	–	–	1.42
Picos	Beech	24	13	–	0.54	0.21	0.08	0.08	0.08	–	–	0.67	0.04	0.08	–	0.17	0.04	0.08
Picos	Mixed	14	13	–	3.43	–	–	0.29	0.14	0.14	–	1.64	0.07	0.14	0.14	0.14	–	0.07
Picos	Oak	9	4	–	0.22	0.22	0.22	–	0.11	0.11	–	0.33	–	–	–	–	–	–
Distrib				NE S	Eur.	Picos	Picos	NWS	Picos	Picos	Orde	Picos	Picos	NW S	Picos	NW S	Picos	S
											F				F			F
											UK				B			UK

*Note*

*n*, number of plots sampled; *n*‐tp, number of plots with terrestrial planarians; Distrib, Species geographical distribution; B, Bulgary; Eur, Europe; F, France; NE, northeast; NW, northwest; Orde, *Ordesa y Monte Perdido* National Park; Picos, *Picos de Europa* National Park; S, Spain; UK, United Kingdom.

### 3.2 Phylogenetic relationships

**Figure 3:**
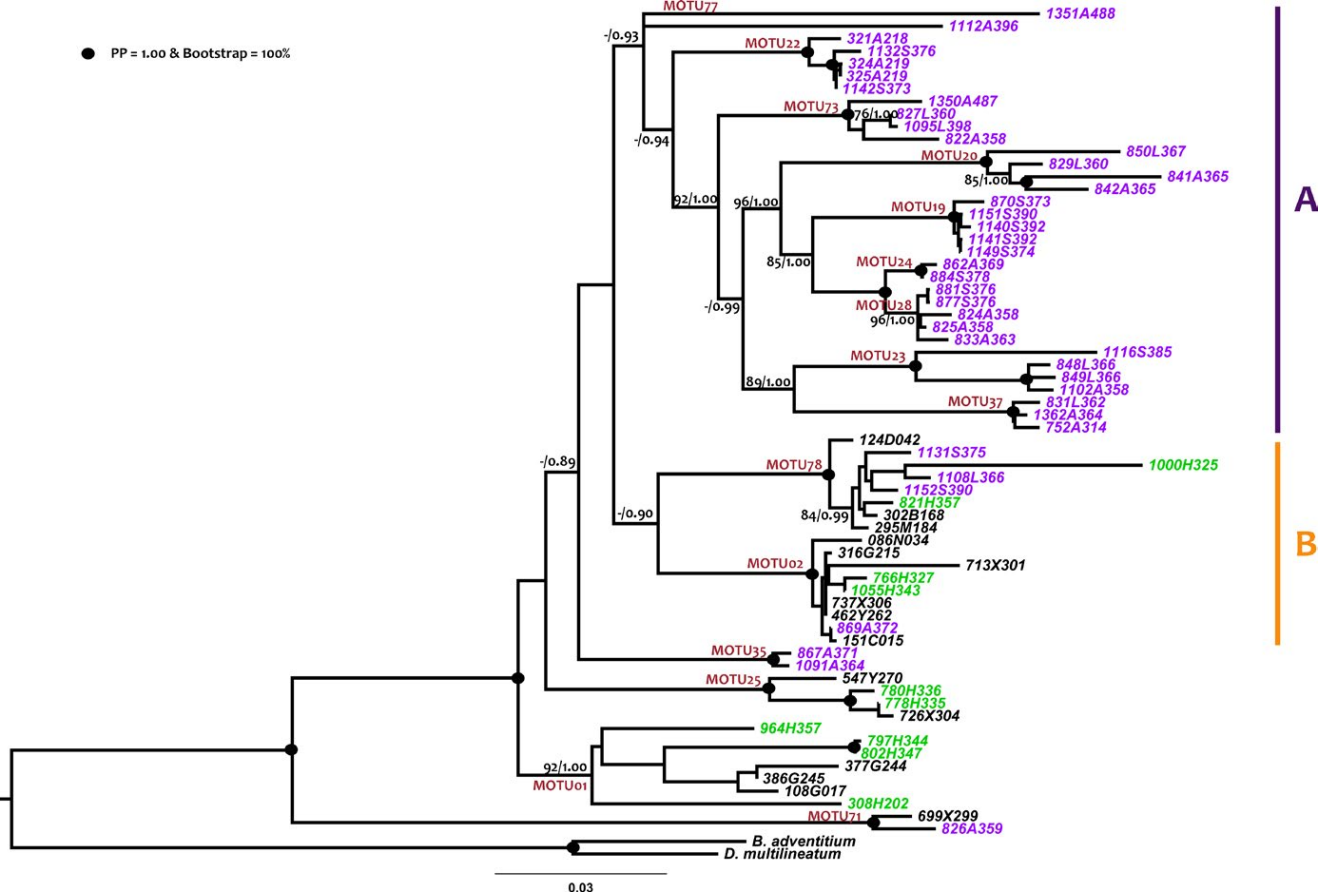
Phylogenetic tree obtained with the concatenated dataset (18S, 28S, and EF). Maximum‐likelihood topology is shown. Numbers at nodes indicate bootstrap (when > 75%) and posterior probability (when > 0.85) values. Black dots at nodes correspond to maximum support values both in bootstrap and posterior probability. A: clade corresponding to MOTUS exclusively from Picos; B: clade corresponding to the species found both in Picos and Ordesa. Scale bar = number of substitutions per site

The analyses of the Cox1 gene phylogeny showed no resolution for the relationships among MOTUs, and the saturation test shows this molecule to be effectively saturated (Supporting information Figure S2); for this reason, only nuclear genes were used to infer the relationships among MOTUs. Figure 3 and Supporting Information Figure S3 show the phylogenetic trees inferred from the concatenated dataset by maximum likelihood and Bayesian inference, respectively; there are small differences between both trees affecting nodes with low support but some clear relationships appear. Both trees show a clade constituted exclusively by MOTUS coming from Picos that is highly supported in BI but not in ML (0.93 PP and 50% BP) (A in Figure 3). This group is sister to a clade (B) constituted by *M. terrestris* (M02) and *M. *cf. *aixandrei*‐2 (M78); the two species present in the two parks and also in other regions around Europe. M35, a species exclusive from Picos, in the BI analyses is sister to clade B, while in the ML tree is sister to clades A and B but with low support in both analyses.

For the rest of species, M71 (from Picos and other European regions) is sister group to all other MOTUS included in the analysis with maximum support. Then, two MOTUS from Ordesa and other regions split, M01 (*M. nana*) followed by M25 (*M. fuscomaculosa*).

**Figure 4:**
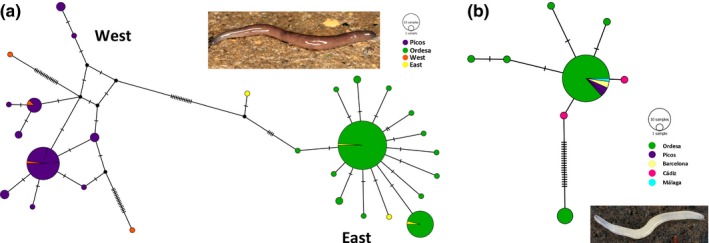
Median‐joining network reconstructed with PopART for the mitochondrial (Cox1) haplotypes: (a) for *Microplana terrestris* (MOTU02), east and west correspond to haplotypes found in the eastern and western clades in Álvarez‐Presas et al. (2012); (b) for *M. cf. aixandrei*‐2 (MOTU78). Circle size is proportional to sample size; crossing lines between haplotypes indicate mutational steps

For *Microplana terrestris* (M02), species shared between parks, the haplotype network (Figure 4a) shows that individuals from Ordesa share haplotypes found in the eastern clade defined in a previous study (Álvarez‐Presas et al., 2012) or have a few differences from those; the haplotypes of the individuals from Picos coincide with haplotypes found in the western clade defined in the same publication. Also as in the previous study, the patterns of diversity between the two clades (east and west) are different, showing a star pattern in the east and a more structured pattern in the west.

As regards the other species shared between parks (Figure 4b), *M. *cf. *aixandrei*‐2 (M78), there is a highly frequent haplotype shared in both parks and also by specimens from other regions of the Iberian Peninsula (Barcelona and Málaga), showing no correlation between genetic structure and geographical distribution at this level. There is an haplotype composed of sequences only from Ordesa highly differentiated from this frequent common haplotype. However, the limited representation of individuals of this species in Picos (only three animals) prevents us from stablishing a good comparison of its haplotype distribution with that of *M. terrestris*.

### 3.3 Abundance, diversity, and community composition at regional scale (parks)

Given the low general abundance of the MOTUs, we have pooled the data from spring and autumn samplings for the following analyses. The proportion of plots without any terrestrial planarian was higher in Picos (34%, 16 of 47 plots) than in Ordesa (11%, five of 46 plots) (Supporting information Table S2). The abundance per MOTU in each plot was in general low with only a few MOTUs reaching values close or over 10 animals in some plots, resulting in mean abundances per type of forest below 1 (Table 1) with a few exceptions (M02, M28, and M78). The distribution of MOTUs is not uniform through parks (Table 1) as demonstrated by the ANOSIM analysis that shows significant differences in species composition between parks (*p* level < 0.05, Table 2).

**Table 2:** Differences in species composition (ANOSIM results) between parks (regional scale) and between forest types (local scale). Data from fall and spring pooled

Comparisons	*R*	*p*
Regional scale		
Ordesa all plots (41) vs Picos all plots (30)	0.256	0.0001*
Local scale by forest type		
Ordesa		
Beech forest (20) vs. Pine forest (21)	0.039	0.1070^ns^
Picos		
Beech forest (13) vs. Oak forest (4) vs. Mixed forest (13)	0.194	0.0040*
Beech forest (13) vs. Oak forest (4)	−0.040	0.6210^ns^
Mixed forest (13) vs. Beech forest (13)	0.192	0.0030*
Mixed forest (13) vs. Oak forest (4)	0.449	0.0200*

*Notes*

ns, not significant probability. *Significant probability.

**Figure 5:**
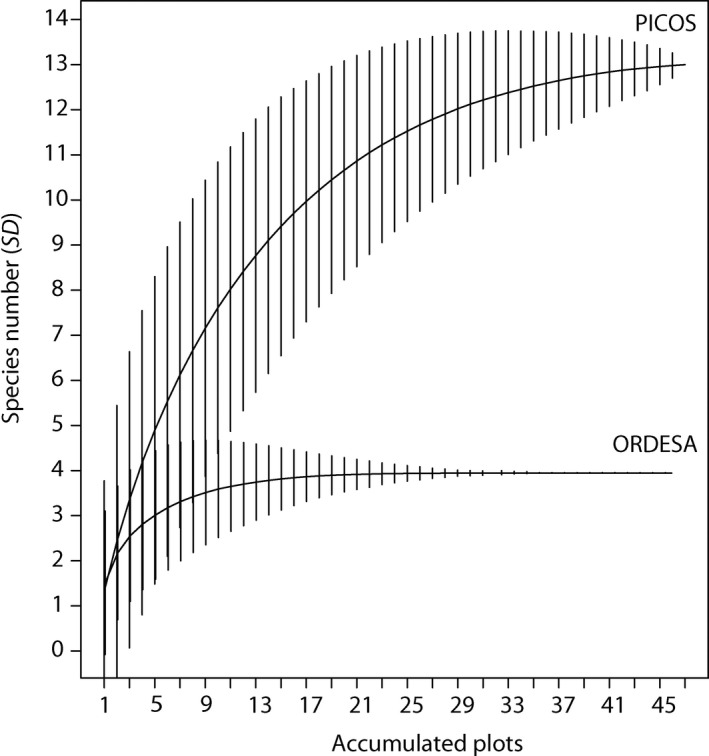
Flatworm species accumulation curves per park. Vertical lines indicate standard deviations

Mean abundances and richness per plot for each park were not significantly different (Table 3). Nonetheless, the total number of species and its diversity was different among parks, presenting Picos a higher richness (13 vs. 4 species, Table 4). The species accumulation models performed with all plots of the two parks (Figure 5) showed that, with around 12 accumulated plots, the predicted species number is higher in Picos than in Ordesa. Also in Ordesa, the predicted species number stabilized quickly (flat line) and with 35 accumulated samples the deviation of the mean is zero, while in Picos, the predicted species number increases continuously with the accumulated plots. Moreover, all three nonparametric total species estimators (Chao‐1, Jack‐1 and Boot) showed a little increment in predicted species number in Picos, but no increment in Ordesa (Table 4). The species accumulation models performed only with beech forests plots of the two parks also show that beech forest in Picos host more species than Ordesa irrespective of the area selected (Supporting information Figure S1). At last, Gamma diversity measured with the Shannon diversity index (H) was higher in Picos than in Ordesa (Table 4).

**Table 3:** Species abundance and richness per plot on each park (regional scale) and forest type within parks (local scale) and Student’s *t* test or ANOVA for the comparisons of abundance and richness

Area	*n*	Ab (*SE*)	Ri (*SE*)
Regional scale			
Ordesa all forests (Ord)	46	4.39 (0.48)	1.57 (0.12)
Picos all forests (Pic)	47	3.15 (0.67)	1.32 (0.18)
Student’s *t*‐test		*t* = 1.499	*t* = 1.135
		*p* = 0.137^ns^	*p* = 0.260^ns^
		Ord = Pic	Ord = Pic
Local scale by forest type			
Ordesa			
Beech forest (Fo)	22	4.05 (0.72)	1.32 (0.14)
Pine forest (P)	24	4.71 (0.66)	1.79 (0.18)
Student’s *t*‐test		*t* = −0680	*t* = −0.473
		*p* = 0.500^ns^	*p* = 0.046*
		Fo = *P*	Fo < *P*
Picos			
Beech forest (Fp)	24	2.08 (0.65)	1.17 (0.26)
Oak forest (Q)	9	1.22 (0.57)	0.78 (0.36)
Mixed forest (M)	14	6.21 (1.68)	1.93 (0.29)
One‐way ANOVA test		*F* = 5.451	*F* = 2.988
		*p* = 0.008*	*p* = 0.061^ns^
		M > Fp = Q	Fp = Q = M

*Notes*

Data from fall and spring pooled. *n*, number of plots; Ab, mean number of individuals per plot; Ri, mean species richness per plot; *SE*, standard error; ns, not significant difference between the above values in the statistic test. *Significant difference between values above.

**Table 4:** Species diversity per parks (regional scale) and per forest type within parks (local scale)

Area	*n*	*S*	*H*	Chao1 (*SD*)	Jack1 (*SD*)	Boot (*SD*)
Regional scale			γ‐*H*			
Ordesa all forests	46	4	1.26	4.00 (0.00)	4.00 (0.00)	4.00 (0.05)
Picos all forests	47	13	2.51	13.17 (0.54)	13.98 (0.98)	13.91 (0.83)
Local scale by forest type			α‐H			
Ordesa						
Beech forest (Fo)	22	4	1.22	4.00 (0.00)	4.00 (0.00)	4.14 (0.34)
Pine forest (P)	24	4	1.28	4.00 (0.00)	4.00 (0.00)	4.05 (0.23)
Picos						
Beech forest (Fp)	24	11	2.81	11.40 (0.87)	12.92 (1.36)	12.41 (1.10)
Oak forest (Q)	9	6	2.48	18.50 (17.14)	10.44 (2.39)	7.84 (1.33)
Mixed forest (M)	14	10	1.96	14.17 (4.88)	14.64 (2.48)	12.13 (1.42)

*Notes*

Data from fall and spring sampling campaigns pooled. *n*, number of plots; *S*, total species number; *H*, Shannon diversity index; γ‐*H*, γ‐diversity measured on each National Park; α‐*H*, α‐diversity measured in each forest type; Chao1, Chao‐1 total species number estimator; Jack1, first‐order jackknife total species number estimator; Boot, bootstrap total species number estimator; *SD*, standard deviation.

### 3.4 Abundance, diversity, and community composition by forest type

**Figure 6:**
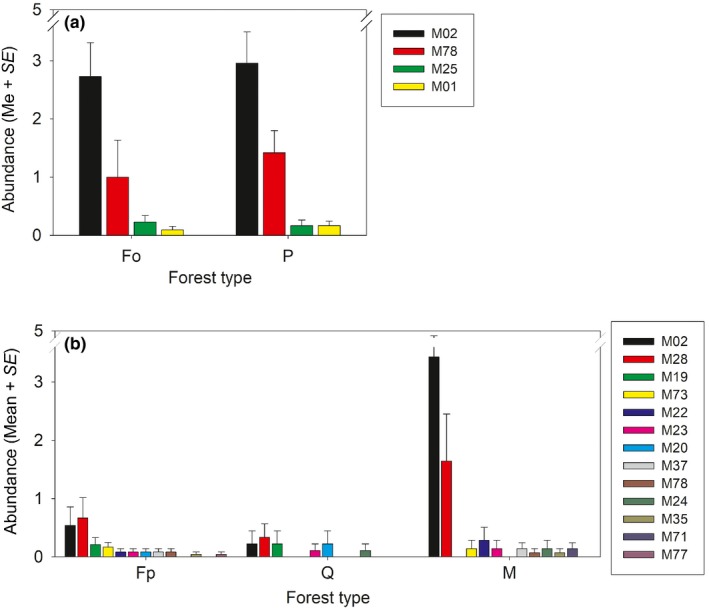
Abundance of flatworm species per plot on each forest type. (a) Ordesa; (b) Picos Forest types: Fo, beech Ordesa; Fp, beech Picos; M, mixed; P, pine; Q, oak.

In the two forest types analyzed in Ordesa, beech, and pine forests, the proportion of plots with terrestrial planarians was high (Table 1). In both forests, the same four species and in the same proportion were found (Figure 6a), and M02 was the most common species, followed by M78 and, further away, by M25 and M01. ANOSIM analysis showed no significant difference in terrestrial flatworm species composition between the two forests (Table 2). In this park, mean terrestrial flatworm’s abundance per plot was similar in the two forest types, while mean richness per plot was significantly higher in pine than in beech forest (Table 3). Total species number (S) was identical in the two forest types (four species), and α‐diversity values measured with Shannon index (H) were also very similar (Table 4). The Sørensen dissimilarity index value (β_*t*_) measured between beech and pine forests in Ordesa was 0.00, meaning that the same species were found in both forest types. As a result, the nonparametric total species estimators showed the same values as actual total species number for beech and pine forests (four species, with only a little deviation for bootstrap estimator, Table 4).

In Picos, three forest types were analyzed, beech forest (11 species of terrestrial flatworms), oak forest (six species), and mixed forest (10 species), the two‐first having a lower proportion of plots with terrestrial planarians (Table 1). In beech and oak forests, the abundance of different species was similar, but in mixed forest, M02 was by far the most abundant species (Figure 6b). ANOSIM analysis (Table 2) showed no significant difference in species composition between beech and oak forests (*p* value > 0.05), but significant differences were found between terrestrial flatworm communities of mixed forest with respect to beech and oak forests (*p* values < 0.05). Mean terrestrial flatworm’s abundance per plot was significantly higher in mixed forest than in beech and oak forests, while mean species richness per plot was not significantly different between these three forest types (Table 3). Shannon diversity values (H) were higher in beech and oak forests than in mixed forest due to a higher abundance of M02 and M28 in the last forest (Table 4, Figure 6b). The mean Sørensen dissimilarity index value (β_*t*_) measured between the three forest types was 0.38 (β_*t*_ between beech and mixed forests = 0.24, between beech and oak forests = 0.41, between mixed and oak forests = 0.50). The three nonparametric species number estimators (Chao1, Jack1 and Boot) showed an increment in total species number in the three forest types compared with actual total species number, especially for oak forest and mixed forest (Table 4).

### 3.5 Environmental parameters

Total annual rainfall and accumulated rainfall of the three previous months to each sampling campaign are shown in Supporting information Table S3. In Ordesa, the mean total annual rainfall for the years of this study was within the normal range of historical data (mean annual rainfall range between 1,129 and 1,690 mm/year). In Picos, however, rainfall was very low during the sampling period (especially in 2013), far below the mean annual precipitation range normal for the park (between 1,109 and 1,968 mm/year) and, which is more important to soil humidity, the accumulated rainfall during the three previous months to sampling dates was also very low (also especially in 2013).

The mean soil water content, temperature, and pH values measured in the two sampling campaigns on every forest type of each national park are shown in Supporting information Table S4. As the same plots were visited during the two sampling campaigns, altitude measures were the same in the two dates (Supporting information Table S4). In Ordesa, in general, beech forest soils showed higher mean water content and pH values and lower temperature than pine forest soils. Comparing the two sampling campaign’ values, the main differences were the higher mean soil water content and the lower mean soil temperature values in 2014 (especially in beech forest). In Picos, generally beech forest soils showed higher mean water content and lower temperature than oak and mixed soil forests, while mean pH value was higher in mixed forest than in beech and oak forests. Comparing the two sampling campaigns values, the main differences were the higher soil water content and temperature, and lower pH values in 2014.

In the two parks, no significant correlation was obtained between flatworm abundance and richness per plot with respect to soil water content and temperature. In Picos, total terrestrial flatworm abundance and richness per plot were significantly correlated with soil pH values (Supporting information Table S2), presenting higher values of abundance and richness those plots with moderate‐to‐high pH values (Supporting information Figure S4), while in Ordesa, these correlations were not significant.

## 4 DISCUSSION

This study presents the first community analysis of terrestrial planarians in Europe. The analysis, performed in two national parks in northern Spain, has revealed an unexpected diversity for this group of animals. However, this diversity is not equivalent in both parks; community composition and total amount of species significantly differ. Moreover, within parks, only Picos shows some significant differences among forest types. In the following sections, we analyze the reasons to explain such differences.

### 4.1 Differences in flatworm’s abundance and richness between forests within parks

Using arthropods as model organism in three national parks in Spain (Aigües Tortes, Ordesa and Picos de Europa), Melguizo‐Ruiz et al. (2012) found higher densities of edaphic arthropod fauna in microsites located at the base of the hillsides, which were more humid and rich in litter. Moreover, these authors point that calcareous soils (with higher pH values) present higher quantities of arthropod edaphic fauna than silicic soils. However, in the case of land planarians, several publications have indicated that some environmental characteristics may affect the presence and abundance of this animal group in forest soils, such as pH, depth, temperature, soil moisture, and prey abundance (Boag, Jones et al., 1998; Boag, Yeated et al., 1998; Fick et al., 2006; Johns et al., 1998; Winsor et al., 1998), but none of them point to any of these factors as the main driver of the presence/absence of these terrestrial invertebrates. Antunes et al. (2012), working in an undisturbed area of *Araucaria* forest, found that terrestrial flatworms were not significantly associated with any particular microhabitat condition, which included environmental parameters as soil humidity, but also leaf‐litter and other fauna composition. Comparing land flatworm communities in two types of forests in Southern Brazil, Fick et al. (2006) found that pH differences together with thermal amplitudes may explain dissimilarities in composition between the communities due to disparities in pH preferences for different species.

In the present study, flatworm abundance and richness per plot showed a significant correlation only with pH in Picos (Supporting information Figure S4), with higher values of these parameters for neutral to basic soils (pH values between 6.5 and 7.5). This could be the explanation of the significant differences found for one of the forest types in Picos, the mixed forest, that seems to harbor higher density of planarians per square meter (individuals per plot), and shows a significantly different species composition with respect to beech and oak forests.

In Ordesa, although the total abundance per plot was equivalent between forests, we found a higher species richness in pine forests. Both forest types in Ordesa showed mean pH values over 6 (Supporting information Table S4), around what appears to be optimal for *Microplana* species attending to the results in Picos (Supporting information Figure S3), which may be one of the factors to explain the lack of differences between beech and pine forests in this park.

### 4.2 Differences in flatworm’s abundance and richness at a regional scale

Our results showed the existence of significant differences between parks in terms of species composition and richness (Table 2) that cannot be explained by differences in the carrying capacity between parks (Table 3), nor by the inequality in the area of the two parks (Supporting information Figure S1). The environmental factors analyzed also fail to explain such differences. However, the historical analysis based on the phylogeny of the species and the genetic structure within the shared species provides some clues about the differences observed.

Of the two shared species between parks, M02, (*M. terrestris*, Figure 4a), presented a highly structured haplotype network with no shared haplotypes between parks. In a previous work (Álvarez‐Presas et al., 2012), we found that *M. terrestris* was divided into two highly differentiated groups in the north of the Iberian peninsula, one east and one west, and hypothesized that the species had followed the forests history of refugia and recolonization during and after the Pleistocene. The western clade (highly genetically structured) may have remained in refugia in the Cantabrian and Basque regions while the eastern clade (presenting only a few very similar haplotypes) may have remained in a very small refuge in the Pyrenees or surrounding area, or even may have arrived from the east or north of Europe after the Last Glacial Maximum (LGM), as it has been hypothesized for some plant species (Hewitt, 1999; Petit et al., 2002). As could be expected, in the present work, the *M. terrestris* haplotypes of the two parks corresponded to the respective geographic clades (Ordesa with the east, west for Picos). In addition, the observed patterns were different, and Picos was highly structured while Ordesa shared haplotypes with populations from more northern localities (UK, France) in a star pattern that is expected for populations that have recently expanded from a small number of individuals (Figure 4a). The present data show the same pattern at a higher taxonomic level: Picos harbored a higher species diversity than Ordesa. Most of the species in Picos are phylogenetically closely related (Figure 3, clade A) and at the same time quite genetically divergent among them. In fact, of all the MOTUs found in this park, only those that are common between parks and with other regions of Europe (M02, M78 and M71) do not belong to the same lineage that apparently diversified in the region. Thus, although we cannot determine exactly the phylogenetic relationships between the endemic MOTUs found in Picos due to the low support in some basal nodes, we can infer an ancient common ancestor for all of them. This scenario could be a consequence of a long process of diversification, not necessarily occurring in the area occupied now by the park. Picos Valleys may have acted as refugia for them during the Pleistocene glaciations, as occurred with *M. terrestris* diversity, and after that they would have remained restricted there, resulting in their present distribution. Factors that may have influenced the fact that these species did not expand from the Picos valleys are probably the inadequacy of the surrounding forests as habitat for them, or their small populations. A deeper analysis will have to be undertaken to test the different hypotheses.

On the other hand, the fact that in Ordesa only four species have been detected, all with a wide distribution range, including the northernmost populations, may reflect, as in the case of *M. terrestris* eastern clade, that this region would have provided a single microrefuge from which M78 and M02 have recolonized Europe. Or, on the contrary, it could have been recently colonized from eastern or northern Europe. Unlike *M. terrestris*,* M. *cf. *aixandrei‐2* is also distributed in the south of the Iberian Peninsula (in Cádiz and Málaga, Mateos et al., 2017) and in this case, the different regions share at least one common haplotype (Figure 4b). This could be indicating a relatively recent expansion, although we cannot know, based on the available data, what was the origin and direction of this colonization, whether from north to south or from south to north, and also if the origin is in the rest of Europe or in the peninsula. We cannot discard, also, the possibility of human transport, although the characteristics of the species and the habitat it occupies (they are very small, white animals that hide under rocks and rotten trunks in humid forests) make it unlikely.

Nonetheless, in both parks, we find species with high genetic diversity and occupying basal positions in the phylogenetic tree, as M71 in Picos and M01 and M25 in Ordesa. These species may represent old lineages with a wide distribution through Europe before the LGM that may have recovered from different refugia throughout the continent.

In summary, historical factors offer plausible explanations to be considered the drivers of the present differences among regions in the Iberian Peninsula.

### 4.3 Final remarks

The species richness found in Ordesa and Picos may seem low if compared with similar studies performed in different Brazilian forests, where total flatworm species number ranges between 18 in areas of deciduous forests (De Castro & Leal‐zanchet, 2005) and 51 in areas of *Araucaria* forest (Leal‐Zanchet et al., 2011). Although these Brazilian species richness result from studies with different sampling effort with respect to our study, both in the number of sampling campaigns (between 14 and 15 in Brazil, two in the present study) and in the number of people involved in the samplings (five collectors in Brazil, two collectors in the present study), the species accumulation models showed both parks are close to a saturation point, indicating there is a realistic species richness difference between the areas involved (temperate Europe vs. neotropical Brazil). Nonetheless, our figures seem extremely high when analyzed on a European context. Till 1998, only 17 species of *Microplana* were known from Europe. Recent works have lately increased the number of species to a total of 43 (Mateos et al., 2017; Sluys et al., 2016; Vila‐farré, Mateos, Sluys, & Romero, 2008; Vila‐Farré, Sluys, Mateos, Jones, & Romero, 2011; Álvarez‐Presas et al. *in preparation*); nonetheless, this is still poor when compared to the more than 119 and 98 recorded species from São Paulo and Florianopolis only in Brazil (Sluys, 1999). These figures simply confirm the known rule that species richness is much higher in the tropics than in temperate and cold regions (Brown, 2014), being terrestrial planarians one more example to certify this fact. Nevertheless, our results confirm another rule stating that there is a very large bias in species descriptions according to the number of taxonomists dedicated to the group: Since we started working on species identification in Europe, we have seen that the number of species has increased rapidly.

We aimed to analyze the terrestrial planarian community composition in two protected areas to have a view of the original fauna situation in front of what can be found out of those areas and to have a first approximation of the value of protected areas for soil communities. Our results allow us to certify that in both parks, terrestrial planarians fauna is representative of the diversity found in their area, and in the case of Picos, it certainly protects a highly endemic and at the same time genetically diverse and genealogically old group of species, which completely fulfills the objectives of the park. Our results also point to the importance of the study of soil fauna, generally poorly considered when planning protected areas management or new protected areas.

## CONFLICT OF INTEREST

None declared.

## AUTHOR CONTRIBUTIONS

The three authors contributed to the design of the project. MAP and EM performed the samplings. MAP was in charge of the laboratory work and analysis of molecular data. EM performed numeric and statistical analyses on communities. The three authors contributed to final analyses, discussion of results, and writing of the ms.
