# Supplementary figures and images for: Hidden diversity in forest soils: Characterization and comparison of terrestrial flatworm’s communities in two national parks in Spain
Source: Ecol Evol. 2018 Jul 2;8(15):7386–400. doi: 10.1002/ece3.4178 (PMC6106173; doi:10.1002/ece3.4178)

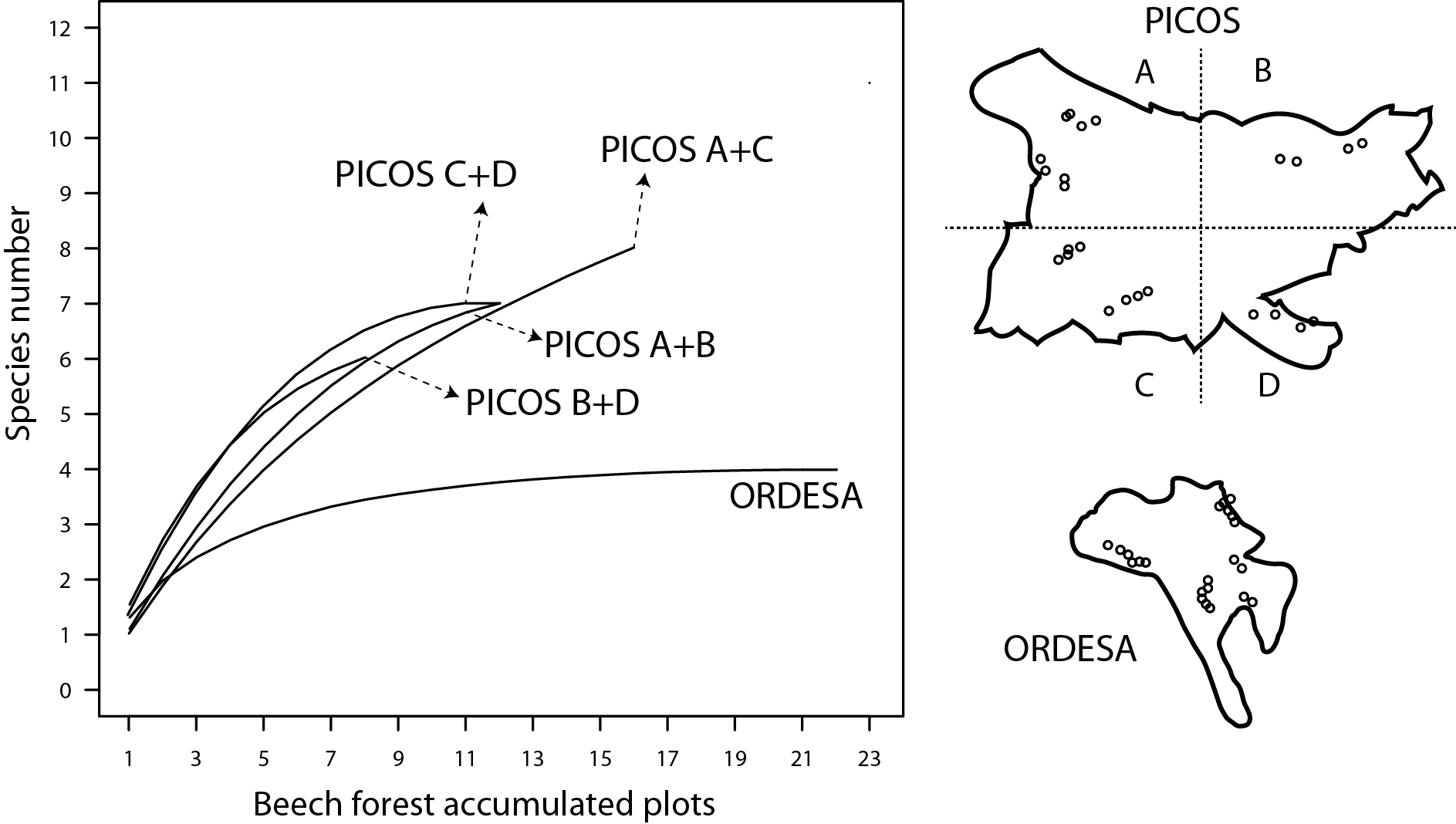

Supplement: Supplementary file 1 [file ECE3-8-7386-s001.tif]

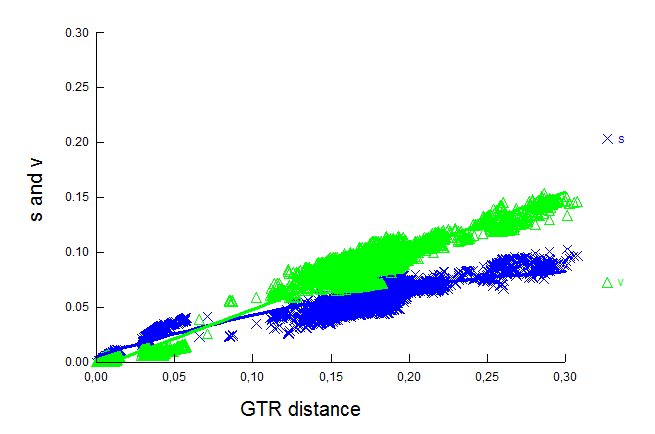

Supplement: Supplementary file 2 [file ECE3-8-7386-s002.tif]

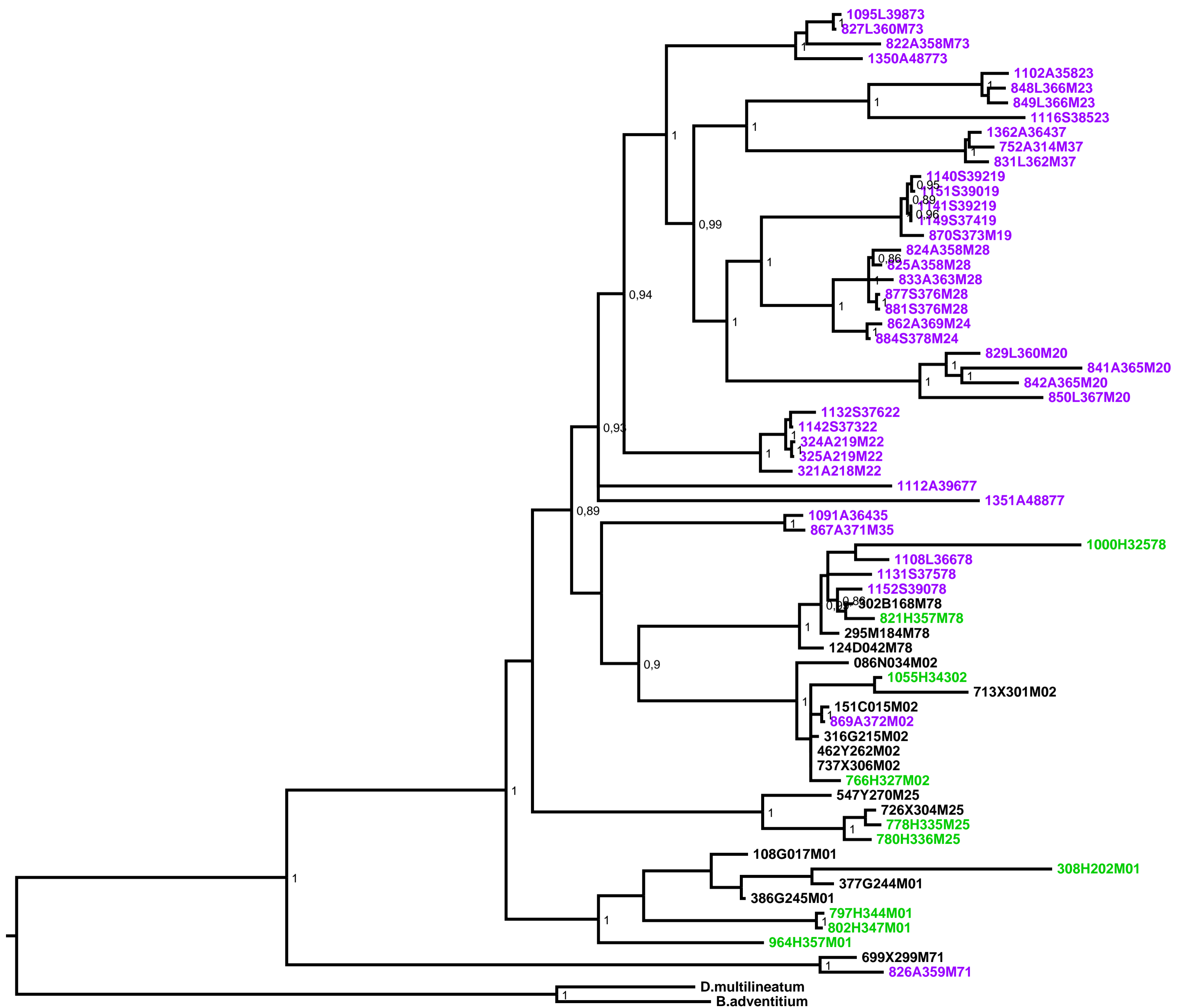

0.03

Supplement: Supplementary file 3 [file ECE3-8-7386-s003.pdf]

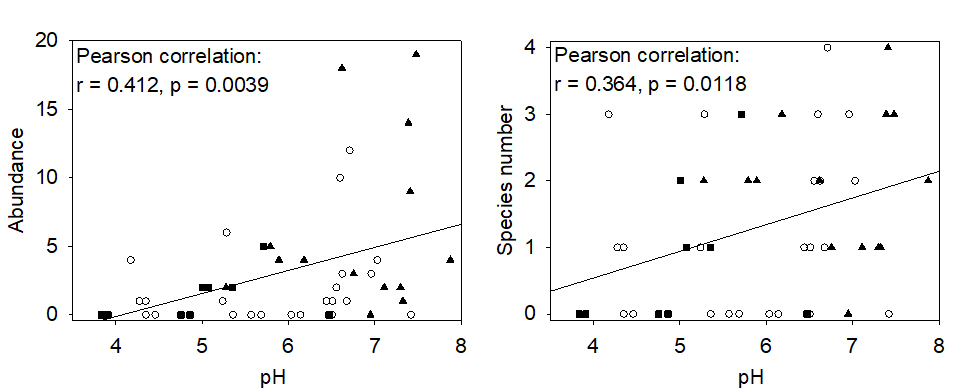

Supplement: Supplementary file 4 [file ECE3-8-7386-s004.tif]
