# Supplementary material for: Hidden diversity in forest soils: Characterization and comparison of terrestrial flatworm’s communities in two national parks in Spain
Source: Ecol Evol. 2018 Jul 2;8(15):7386–400. doi: 10.1002/ece3.4178 (PMC6106173; doi:10.1002/ece3.4178)
Supplement: Supplementary file 5 [file ECE3-8-7386-s005.pdf]

Supporting information table S1. List of specimens used in the molecular analyses. Species and corresponding MOTU in Cox1 phylogeny, locality of origin, plot code (in case of Ordesa and Picos localities), codes used in the trees, and accession numbers for the gene sequences.

Species	Collecting locality	Plot code	Molecular code	GenBank			
				Cox1	18S	28S	EF
Microplana cf. *aixandrei-2* MOTU78	Huesca, Spain (Ordesa)	PI02	756H324 [B]	KU867105 [C]			
		PI04	758H325 [B]	MH056878* [C]			
		PI04	759H325 [B]	MH056879* [C]			
		PI08	783H337 [B]	MH056898* [C]			
		ES06	785H339 [B]	KU867106 [C]			
		ES08	788H340 [B]	MH056899* [C]			
		AN06	799H345 [B]	MH056908* [C]			
		ES10	808H349 [B]	MH056914* [C]			
		AN12	821H357 [B]	KU867107 [C]	KU872576	KU872618	KU872662
		PI08	953H337 [B]	MH056996* [C]			
		AN07	955H350 [B]	MH056997* [C]			
		AN08	960H351 [B]	MH057002* [C]			
		AN12	961H357 [B]	MH057003* [C]			
		AN12	962H357 [B]	MH057004* [C]			
		OR04	977H331 [B]	MH057016* [C]			
		PI03	990H412 [B]	MH057029* [C]			
		PI04	991H325 [B]	MH057030* [C]			
		PI04	992H325 [B]	MH057031* [C]			
		PI04	993H325 [B]	MH057032* [C]			
		PI04	994H325 [B]	MH057033* [C]			
		PI04	995H325 [B]	MH057034* [C]			
		PI04	996H325 [B]	MH057035* [C]			
		PI04	998H325 [B]	MH057036* [C]			
		PI04	999H325 [B]	MH057037* [C]			
		PI04	1000H325 [B]	KU867108 [C]	KU872577	-	KU872663
		PI04	1001H325 [B]	MH056726* [C]			
		PI04	1002H325 [B]	KU867109 [C]			
		ES04	1005H328 [B]	MH056729* [C]			
		ES04	1006H328 [B]	MH056730* [C]			

		ES08	1010H340[B]	MH056734*[C]			
		ES08	1011H340[B]	KU867110[C]			
		ES10	1014H349[B]	MH056737*[C]			
		ES10	1015H349[B]	MH056738*[C]			
		OR01	1023H416[B]	MH056745*[C]			
		PI09	1034H346[B]	MH056756*[C]			
		PI09	1035H346[B]	MH056757*[C]			
		PI09	1036H346[B]	MH056758*[C]			
		PI10	1039H414[B]	MH056760*[C]			
		PI10	1040H414[B]	MH056761*[C]			
		PI10	1041H414[B]	MH056762*[C]			
		PI10	1042H414[B]	MH056763*[C]			
		PI10	1043H414[B]	MH056764*[C]			
		PI10	1044H414[B]	MH056765*[C]			
		AN06	1061H345[B]	MH056780*[C]			
		AN10	1064H408[B]	MH056782*[C]			
		AN10	1065H408[B]	MH056783*[C]			
		AN10	1066H408[B]	MH056784*[C]			
		OR09	1077H352[B]	MH056794*[C]			
	León, Spain (Picos)	VA08	1108L366[B]	MH056825*[C]	MH056662*	-	MH057042*
	Cantabria, Spain (Picos)	DE04	1131S375[B]	MH056848*[C]	MH056665*	MH056699*	MH057045*
		DE03	1152S390[B]	MH056864*[C]	MH056670*	MH056704*	MH057050*
	Barcelona, Spain		254B005[B]	KU867090			
			302B168[B]	KU867093	KU872572	KU872616	KU872659
	Cádiz, Spain		124D042		KU872574	KU872617	KU872661
			284D183[B]	KU867100			
			285D183[B]	KU867101			
	Málaga, Spain		295M184[B]	KU867103	KU872575	-	-
Microplana fuscomaculosa	Cumbria, United Kingdom		547Y270		KU872584	KU872623	KU872670
MOTU25	Le Chipal, France		726X304		KU872586	-	KU872672
	Huesca, Spain (Ordesa)	PI05	778H335	KU867153[C]	KU872587	KU872624	KU872673
		PI07	780H336	KU867154[C]	KU872588	KU872625	KU872674
		ES05	784H338	KU867155[C]			
		ES06	786H339	KU867156[C]			
		PI09	801H346	KU867157[C]			
		PI12	804H348	KU867158[C]			

		PI12	954H348	KU867159 [C]			
		AN01	1018H332	KU867160 [C]			
		PI07	1050H336	KU867161 [C]			
Microplana nana	Girona, Spain		108G017		FJ969981	KJ599722	-
MOTU01			377G244		KR906659	KU872635	KU872687
			386G245		KR906660	-	-
	Huesca, Spain		308H202		-	KU872636	KU872688
	Huesca, Spain (Ordesa)	AN02	774H333	KR906598 [C]			
		AN05	797H344	KR906599 [C]	KR906661	-	-
		PI11	802H347	KR906600 [C]	KR906662	KU872637	KU872689
		AN09	819H355	KR906601 [C]			
		AN12	964H357	KR906602 [C]	KR906663	-	-
		PI07	1049H336	MH056769* [C]			
Microplana nervosa	Asturias, Spain		324A219		KU872598	KU872638	KU872690
MOTU22			325A219		KU872599	-	KU872691
	Asturias, Spain (Picos)	LA08	868A371	KU867191 [C]			
	Cantabria, Spain (Picos)	DE08	1132S376	KU867192 [C]	KU872600	KU872640	KU872692
		DE08	1133S376	KU867193 [C]			
		DE08	1134S376	KU867194 [C]			
		DE01	1142S373	KU867195 [C]	KU872601	-	KU872693
		DE02	1145S374	KU867196 [C]			
Microplana sp. 1	Saint Laurent du Pont, France		699X299		KU872602	KU872643	KU872696
MOTU71	Asturias, Spain (Picos)	LA04	826A359	KU867198 [C]	KU872603	KU872644	-
		BE08	885A379	KU867230 [C]			
Microplana sp. 5	Asturias, Spain		321A218		KU872607	KU872649	KU872700
Microplana sp.	Cantabria, Spain (Picos)	DE01	870S373	MH056970* [C]	MH056690*	MH056722*	MH057069*
MOTU19		DE06	1140S392	MH056854* [C]	MH056666*	MH056700*	MH057046*
		DE06	1141S392	MH056855* [C]	MH056667*	MH056701*	MH057047*
		DE02	1144S374	MH056857* [C]			
		DE02	1149S374	MH056861* [C]	MH056668*	MH056702*	MH057048*
		DE03	1150S390	MH056862* [C]			
		DE03	1151S390	MH056863* [C]	MH056669*	MH056703*	MH057049*
Microplana sp.	León, Spain (Picos)	SA02	829L360	MH056932* [C]	MH056679*	MH056712*	MH057058*
MOTU20		SA05	850L367	MH056953* [C]	MH056686*	MH056719*	MH057065*
	Asturias, Spain (Picos)	AM06	841A365	MH056944* [C]	MH056682*	MH056715*	MH057061*
		AM06	842A365	MH056945* [C]	MH056683*	MH056716*	MH057062*

Microplana sp.	Asturias, Spain (Picos)	BE05	854A369	MH056956* C			
MOTU24		BE05	862A369	MH056964* C	MH056687*	MH056720*	MH057066*
	Cantabria, Spain (Picos)	BE06	884S378	MH056984* C	MH056693*	MH056725*	MH057072*
Microplana sp.	Asturias, Spain (Picos)	LA01	823A358	MH056927* C			
MOTU28		LA01	824A358	MH056928* C	MH056676*	MH056710*	MH057056*
		LA01	825A358	MH056929* C	MH056677*	-	MH057057*
		AM01	832A363	MH056935* C			
		AM01	833A363	MH056936* C	MH056681*	MH056714*	MH057060*
		AM02	836A364	MH056939* C			
		AM02	837A364	MH056940* C			
		AM02	839A364	MH056942* C			
		AM06	843A365	MH056946* C			
		AM06	844A365	MH056947* C			
		BE05	852A369	MH056955* C			
		BE05	856A369	MH056958* C			
		BE05	860A369	MH056962* C			
		BE05	863A369	MH056965* C			
		AM01	1085A363	MH056802* C			
		AM02	1086A364	MH056803* C			
		AM02	1087A364	MH056804* C			
		AM02	1088A364	MH056805* C			
		AM02	1092A364	MH056809* C			
		LA01	1103A358	MH056820* C			
		BE05	1118A369	MH056835* C			
		BE05	1119A369	MH056836* C			
		BE05	1120A369	MH056837* C			
	Cantabria, Spain (Picos)	DE08	876S376	MH056976* C			
		DE08	877S376	MH056977* C	MH056691*	MH056723*	MH057070*
		DE08	878S376	MH056978* C			
		DE08	879S376	MH056979* C			
		DE08	880S376	MH056980* C			
		DE08	881S376	MH056981* C	MH056692*	MH056724*	MH057071*
		BE06	883S378	MH056983* C			
		DE08	1135S376	MH056849* C			
		DE08	1137S376	MH056851* C			
	León, Spain (Picos)	SA02	828L360	MH056931* C			

		VA08	845L366	MH056948* C			
		VA08	846L366	MH056949* C			
		SA07	1099L368	MH056816* C			
		SA07	1100L368	MH056817* C			
		VA08	1109L366	MH056826* C			
		VA08	1110L366	MH056827* C			
		VA08	1111L366	MH056828* C			
		SA02	1113L360	MH056830* C			
Microplana sp.	Asturias, Spain (Picos)	LA08	867A371	MH056968* C	MH056688*	MH056721*	MH057067*
MOTU35		AM02	1091A364	MH056808* C	MH056659*	MH056694*	MH057039*
Microplana sp.	Asturias, Spain		752A314	MH069206* C	MH056673*	MH056707*	MH057053*
MOTU37			1362A364	MH056876* C	MH056672*	MH056706*	MH057052*
	Asturias, Spain (Picos)	AM02	1089A364	MH056806* C			
		AM04	1094A382	MH056811* C			
		BE03	1117A387	MH056834* C			
	León, Spain (Picos)	SA04	831L362	MH056934* C	MH056680*	MH056713*	MH057059*
		VA08	847L366	MH056950* C			
Microplana sp.	Asturias, Spain		1350A487	MH056874* C	-	MH056705*	MH057051*
MOTU73	Asturias, Spain (Picos)	AM07	1125A372	MH056842* C			
		AM07	1126A372	MH056843* C			
	León, Spain (Picos)	LA01	822A358	MH056926* C	MH056675*	MH056709*	MH057055*
		SA02	827L360	MH056930* C	MH056678*	MH056711*	-
		AM04	1093A382	MH056810* C			
		SA01	1095L398	MH056812* C	MH056660*	MH056695*	MH057040*
Microplana sp.	Asturias, Spain (Picos)	LA05	1112A396	MH056829* C	MH056663*	MH056697*	MH057043*
MOTU77	Asturias, Spain		1351A488	MH056875* C	MH056671*	-	-
Microplana terrestris	Navarra, Spain		086N034 A	FJ969951	KU872610	KU872651	-
MOTU02			215N061 A	JN379975			
	Huesca, Spain		303H200 A	JN379991			
			306H200 A	JN379993			
	Huesca, Spain (Ordesa)	PI02	757H324 A	MH056877* C			
		PI04	760H325 A	MH056880* C			
		ES01	761H326 A	MH056881* C			
		ES01	762H326 A	MH056882* C			
		ES01	763H326 A	MH056883* C			
		ES01	764H326 A	MH056884* C			

ES02	765H327[A]	MH056885*[C]			
ES02	766H327[A]	MH056886*[C]	MH056674*	MH056708*	MH057054*
ES04	767H328[A]	MH056887*[C]			
OR02	768H329[A]	MH056888*[C]			
OR03	769H330[A]	MH056889*[C]			
OR04	771H331[A]	KR906642[C]			
AN01	772H332[A]	MH056890*[C]			
AN01	773H332[A]	MH056891*[C]			
AN02	775H333[A]	MH056892*[C]			
AN03	776H334[A]	MH056893*[C]			
AN03	777H334[A]	MH056894*[C]			
PI05	779H335[A]	MH056895*[C]			
PI07	781H336[A]	MH056896*[C]			
PI08	782H337[A]	MH056897*[C]			
ES08	787H340[A]	KR906643[C]			
OR06	789H341[A]	MH056900*[C]			
OR06	790H341[A]	MH056901*[C]			
OR06	791H341[A]	MH056902*[C]			
OR06	792H341[A]	KR906644[C]			
OR07	793H342[A]	MH056903*[C]			
OR07	794H342[A]	MH056904*[C]			
AN04	795H343[A]	MH056905*[C]			
AN04	796H343[A]	MH056906*[C]			
AN05	798H344[A]	MH056907*[C]			
AN06	800H345[A]	MH056909*[C]			
PI11	803H347[A]	MH056910*[C]			
PI12	805H348[A]	MH056911*[C]			
PI12	806H348[A]	MH056912*[C]			
ES10	807H349[A]	MH056913*[C]			
AN07	809H350[A]	MH056915*[C]			
AN08	810H351[A]	MH056916*[C]			
AN08	811H351[A]	MH056917*[C]			
AN08	812H351[A]	MH056918*[C]			
AN08	813H351[A]	MH056919*[C]			
OR09	814H352[A]	MH056920*[C]			
OR09	815H352[A]	MH056921*[C]			

OR11	816H353 [A]	MH056922* [C]
OR11	817H353 [A]	MH056923* [C]
OR12	818H354 [A]	MH056924* [C]
AN11	820H356 [A]	MH056925* [C]
PI05	949H335 [A]	MH056993* [C]
PI06	950H413 [A]	MH056994* [C]
PI06	951H413 [A]	MH056995* [C]
AN07	956H350 [A]	MH056998* [C]
AN08	957H351 [A]	MH056999* [C]
AN08	958H351 [A]	MH057000* [C]
AN08	959H351 [A]	MH057001* [C]
AN12	965H357 [A]	MH057005* [C]
AN12	966H357 [A]	MH057006* [C]
AN12	967H357 [A]	MH057007* [C]
OR03	968H330 [A]	MH057008* [C]
OR03	969H330 [A]	MH057009* [C]
OR03	970H330 [A]	MH057010* [C]
OR03	971H330 [A]	MH057011* [C]
OR03	972H330 [A]	MH057012* [C]
OR03	973H330 [A]	MH057013* [C]
OR03	974H330 [A]	MH057014* [C]
OR03	975H330 [A]	MH057015* [C]
OR04	978H331 [A]	MH057017* [C]
OR11	979H353 [A]	MH057018* [C]
OR11	980H353 [A]	MH057019* [C]
OR12	981H354 [A]	MH057020* [C]
OR12	982H354 [A]	MH057021* [C]
OR12	983H354 [A]	MH057022* [C]
ES01	984H326 [A]	MH057023* [C]
ES01	985H326 [A]	MH057024* [C]
ES01	986H326 [A]	MH057025* [C]
ES07	987H410 [A]	MH057026* [C]
ES07	988H410 [A]	MH057027* [C]
PI02	989H324 [A]	MH057028* [C]
PI05	1003H335 [A]	MH056727* [C]
ES02	1004H327 [A]	MH056728* [C]

ES04	1007H328[A]	MH056731*[C]			
ES04	1008H328[A]	MH056732*[C]			
ES04	1009H328[A]	MH056733*[C]			
ES08	1012H340[A]	MH056735*[C]			
ES08	1013H340[A]	MH056736*[C]			
ES10	1016H349[A]	MH056739*[C]			
AN01	1017H332[A]	MH056740*[C]			
AN01	1019H332[A]	MH056741*[C]			
AN01	1020H332[A]	MH056742*[C]			
AN01	1021H332[A]	MH056743*[C]			
AN01	1022H332[A]	MH056744*[C]			
OR06	1024H341[A]	MH056746*[C]			
OR06	1025H341[A]	MH056747*[C]			
OR06	1026H341[A]	MH056748*[C]			
OR06	1027H341[A]	MH056749*[C]			
OR05	1028H417[A]	MH056750*[C]			
OR02	1029H329[A]	MH056751*[C]			
OR02	1030H329[A]	MH056752*[C]			
OR02	1031H329[A]	MH056753*[C]			
OR02	1032H329[A]	MH056754*[C]			
OR02	1033H329[A]	MH056755*[C]			
PI09	1037H346[A]	MH056759*[C]			
PI10	1046H414[A]	MH056766*[C]			
PI10	1047H414[A]	MH056767*[C]			
PI11	1048H347[A]	MH056768*[C]			
AN04	1051H343[A]	MH056770*[C]			
AN04	1052H343[A]	MH056771*[C]			
AN04	1053H343[A]	MH056772*[C]			
AN04	1054H343[A]	MH056773*[C]			
AN04	1055H343[A]	MH056774*[C]	-	-	MH057038*
AN05	1056H344[A]	MH056775*[C]			
AN05	1057H344[A]	MH056776*[C]			
AN05	1058H344[A]	MH056777*[C]			
AN06	1059H345[A]	MH056778*[C]			
AN06	1060H345[A]	MH056779*[C]			
AN09	1062H355[A]	MH056781*[C]			

	AN10	1067H408 [A]	MH056785* [C]			
	AN10	1068H408 [A]	MH056786* [C]			
	AN10	1069H408 [A]	MH056787* [C]			
	AN10	1070H408 [A]	MH056788* [C]			
	AN10	1071H408 [A]	MH056789* [C]			
	AN10	1072H408 [A]	MH056790* [C]			
	AN11	1074H356 [A]	MH056791* [C]			
	AN11	1075H356 [A]	MH056792* [C]			
	AN11	1076H356 [A]	MH056793* [C]			
	OR09	1078H352 [A]	MH056795* [C]			
	OR09	1079H352 [A]	MH056796* [C]			
	OR09	1080H352 [A]	MH056797* [C]			
	OR09	1081H352 [A]	MH056798* [C]			
	OR09	1082H352 [A]	MH056799* [C]			
	OR09	1083H352 [A]	MH056800* [C]			
	OR09	1084H352 [A]	MH056801* [C]			
Asturias, Spain		322A218 [A]	JN380003			
Asturias, Spain (Picos)	AM01	834A363 [A]	MH056937* [C]			
	AM02	835A364 [A]	MH056938* [C]			
	AM02	838A364 [A]	MH056941* [C]			
	BE05	855A369 [A]	MH056957* [C]			
	BE05	857A369 [A]	MH056959* [C]			
	BE05	858A369 [A]	MH056960* [C]			
	BE05	859A369 [A]	MH056961* [C]			
	BE05	861A369 [A]	MH056963* [C]			
	LA07	864A370 [A]	MH056966* [C]			
	LA07	865A370 [A]	MH056967* [C]			
	AM07	869A372 [A]	MH056969* [C]	MH056689*	-	MH057068*
	BE08	886A379 [A]	MH056985* [C]			
	BE08	887A379 [A]	MH056986* [C]			
	BE08	888A379 [A]	MH056987* [C]			
	BE08	889A379 [A]	MH056988* [C]			
	BE08	890A379 [A]	MH056989* [C]			
	BE08	891A380 [A]	MH056990* [C]			
	BE08	892A380 [A]	MH056991* [C]			
	BE08	893A380 [A]	MH056992* [C]			

	AM02	1090A364 [A]	MH056807* [C]
	LA03	1104A395 [A]	MH056821* [C]
	LA04	1105A359 [A]	MH056822* [C]
	BE05	1121A369 [A]	MH056838* [C]
	BE05	1122A369 [A]	MH056839* [C]
	BE05	1123A369 [A]	MH056840* [C]
	BE05	1124A369 [A]	MH056841* [C]
	AM07	1127A372 [A]	MH056844* [C]
	LA08	1128A371 [A]	MH056845* [C]
	LA08	1129A371 [A]	MH056846* [C]
	BE08	1153A379 [A]	MH056865* [C]
	BE08	1154A379 [A]	MH056866* [C]
	BE08	1155A379 [A]	MH056867* [C]
	BE08	1156A379 [A]	MH056868* [C]
	BE08	1157A379 [A]	MH056869* [C]
	BE08	1158A379 [A]	MH056870* [C]
	BE08	1159A379 [A]	MH056871* [C]
	BE08	1160A379 [A]	MH056872* [C]
	BE08	1161A379 [A]	MH056873* [C]
Cantabria, Spain (Picos)	DE02	871S374 [A]	MH056971* [C]
	DE02	872S374 [A]	MH056972* [C]
	DE02	873S374 [A]	MH056973* [C]
	DE02	874S374 [A]	MH056974* [C]
	DE04	875S375 [A]	MH056975* [C]
	DE09	882S377 [A]	MH056982* [C]
	DE09	1130S377 [A]	MH056847* [C]
	DE08	1136S376 [A]	MH056850* [C]
	DE08	1138S376 [A]	MH056852* [C]
	DE08	1139S376 [A]	MH056853* [C]
	DE01	1143S373 [A]	MH056856* [C]
	DE02	1146S374 [A]	MH056858* [C]
	DE02	1147S374 [A]	MH056859* [C]
	DE02	1148S374 [A]	MH056860* [C]
León, Spain (Picos)	SA04	830L362 [A]	MH056933* [C]
	SA07	851L368 [A]	MH056954* [C]
	SA03	1096L361 [A]	MH056813* [C]

		SA03	1097L361 [A]	MH056814* [C]			
		SA07	1098L368 [A]	MH056815* [C]			
		SA07	1101L368 [A]	MH056818* [C]			
		VA04	1106L404 [A]	MH056823* [C]			
		VA04	1107L404 [A]	MH056824* [C]			
		SA04	1114L362 [A]	MH056831* [C]			
		SA04	1115L362 [A]	MH056832* [C]			
	Girona, Spain		269G215 [A]	JN379985			
			316G215 [A]	JN380001	KU872611	KU872653	KU872703
	Buckinghamshire, United Kingdom		462Y262		KR906682	KU872654	KU872704
	A Coruña, Spain		151C015 [A]	FJ969952	FJ969983	KJ599724	KU872705
	La Châtelaine, France		713X301		KU872612	KU872656	KU872707
	Auliac Dessús, France		737X306		KU872613	KU872657	KU872708
Microplaninae sp.	Asturias, Spain (Picos)	AM06	840A365	MH056943* [C]			
MOTU23		LA01	1102A358	MH056819* [C]	MH056661*	MH056696*	MH057041*
	León, Spain (Picos)	VA08	848L366	MH056951* [C]	MH056684*	MH056717*	MH057063*
		VA08	849L366	MH056952* [C]	MH056685*	MH056718*	MH057064*
	Cantabria, Spain (Picos)	BE01	1116S385	MH056833* [C]	MH056664*	MH056698*	MH057044*
**Outgroup**							
Bipalium adventitium	Kingston, USA		*B. adventitium*	AF178306 [C]	DQ666000	DQ665956	KJ599681
Diversibipalium multilineatum	Leguevin, France		*D. multilineatum*	KT922162 [C]			
Diversibipalium multilineatum	Naka, Nagaoka, Niigata, Japan		*D. multilineatum*		DQ666012	DQ665957	KU872709

* Sequences from this study

[A] M02 Network dataset

[B] M78 Network dataset

[C] Cox1 dataset

Supporting information table S2. pH values, forest type and terrestrial flatworm abundance on each plot (data from spring and fall pooled). Mxx refers to the MOTU number (see main text). Types of forest: Fo – beech forest in Ordesa, Fp – beech forest in Picos, M – mixed forest, P – pine forest, Q – oak forest, - MOTU not found.

A) Ordesa

Plot code	Forest	pH	M-1	M-2	M25	M78
AN01	Fo	7.92	-	7	1	-
AN02	Fo	6.56	1	1	-	-
AN03	Fo	6.89	-	2	-	-
AN04	Fo	5.98	-	7	-	-
AN05	Fo	6.26	1	4	-	-
AN06	Fo	6.48	-	3	-	2
ES01	Fo	6.91	-	7	-	-
ES03	Fo	7.28	-	-	-	-
ES05	Fo	6.85	-	-	1	-
ES06	Fo	7.02	-	-	1	1
OR01	Fo	7.62	-	-	-	1
OR02	Fo	7.46	-	6	-	-
OR05	Fo	7.51	-	1	-	-
OR06	Fo	7.38	-	8	-	-
OR11	Fo	6.40	-	4	-	-
OR12	Fo	7.81	-	4	-	-
PI01	Fo	5.71	-	-	-	-
PI02	Fo	6.64	-	2	-	1
PI03	Fo	5.97	-	-	-	1
PI04	Fo	6.62	-	1	-	14
PI08	Fo	5.88	-	1	-	2
PI12	Fo	6.53	-	2	2	-
AN07	P	6.19	-	2	-	1
AN08	P	6.53	-	7	-	1
AN09	P	5.93	1	1	-	1
AN10	P	5.54	-	6	-	3
AN11	P	7.03	-	4	-	1
AN12	P	6.54	1	3	-	4
ES02	P	6.92	-	3	-	-
ES04	P	6.28	-	4	-	2
ES07	P	6.24	-	2	-	-
ES08	P	6.49	-	3	-	3
ES09	P	5.85	-	-	-	-
ES10	P	6.51	-	2	-	3
OR03	P	5.33	-	1-	-	1
OR04	P	6.38	-	2	-	1
OR07	P	6.06	-	2	-	-
OR08	P	4.95	-	-	-	-
OR09	P	5.53	-	9	-	1
OR10	P	5.20	-	-	-	-
PI05	P	7.34	-	3	1	-
PI06	P	6.66	-	2	-	1
PI07	P	5.44	1	1	2	-
PI09	P	4.76	-	1	1	3
PI10	P	5.58	-	2	-	8
PI11	P	5.93	1	2	-	-

B) Picos

Plot	Forest	pH	M02	M19	M20	M22	M23	M24	M28	M35	M37	M71	M73	M77	M78
AM01	Fp	7.03	1	-	-	-	-	-	3	-	-	-	-	-	-
AM02	Fp	6.71	3	-	-	-	-	-	7	1	1	-	-	-	-
AM03	Fp	6.51	-	-	-	-	-	-	-	-	-	-	-	-	-
AM04	Fp	6.51	-	-	-	-	-	-	-	-	-	-	1	-	-
BE01	Fp	6.68	-	-	-	-	1	-	-	-	-	-	-	-	-
BE02	Fp	6.04	-	-	-	-	-	-	-	-	-	-	-	-	-
BE03	Fp	6.44	-	-	-	-	-	-	-	-	1	-	-	-	-
BE04	Fp	7.42	-	-	-	-	-	-	-	-	-	-	-	-	-
DE01	Fp	6.96	1	1	-	1	-	-	-	-	-	-	-	-	-
DE02	Fp	6.60	7	2	-	1	-	-	-	-	-	-	-	-	-
DE03	Fp	6.63	-	2	-	-	-	-	-	-	-	-	-	-	1
DE04	Fp	6.56	1	-	-	-	-	-	-	-	-	-	-	-	1
LA01	Fp	5.29	-	-	-	-	1	-	4	-	-	-	1	-	-
LA02	Fp	5.57	-	-	-	-	-	-	-	-	-	-	-	-	-
LA05	Fp	5.24	-	-	-	-	-	-	-	-	-	-	-	1	-
LA06	Fp	6.14	-	-	-	-	-	-	-	-	-	-	-	-	-
SA01	Fp	4.28	-	-	-	-	-	-	-	-	-	-	1	-	-
SA02	Fp	4.18	-	-	1	-	-	-	2	-	-	-	1	-	-
SA05	Fp	4.35	-	-	1	-	-	-	-	-	-	-	-	-	-
SA06	Fp	4.46	-	-	-	-	-	-	-	-	-	-	-	-	-
VA01	Fp	4.35	-	-	-	-	-	-	-	-	-	-	-	-	-
VA02	Fp	5.36	-	-	-	-	-	-	-	-	-	-	-	-	-
VA05	Fp	3.90	-	-	-	-	-	-	-	-	-	-	-	-	-
VA06	Fe	5.69	-	-	-	-	-	-	-	-	-	-	-	-	-
BE05	M	7.48	9	-	-	-	-	2	8	-	-	-	-	-	-
BE08	M	6.62	17	-	-	-	-	-	-	-	-	1	-	-	-
DE05	M	6.95	-	-	-	-	-	-	-	-	-	-	-	-	-
DE08	M	7.39	3	-	-	3	-	-	8	-	-	-	-	-	-
DE09	M	7.30	2	-	-	-	-	-	-	-	-	-	-	-	-
SA04	M	5.89	3	-	-	-	-	-	-	-	1	-	-	-	-
SA07	M	5.79	3	-	-	-	-	-	2	-	-	-	-	-	-
VA04	M	7.11	2	-	-	-	-	-	-	-	-	-	-	-	-
VA08	M	7.41	-	-	-	-	2	-	5	-	1	-	-	-	1
AM07	M	7.88	2	-	-	-	-	-	-	-	-	-	2	-	-
LA03	M	7.33	1	-	-	-	-	-	-	-	-	-	-	-	-
LA04	M	5.28	1	-	-	-	-	-	-	-	-	1	-	-	-
LA07	M	6.76	3	-	-	-	-	-	-	-	-	-	-	-	-
LA08	M	6.18	2	-	-	1	-	-	-	1	-	-	-	-	-
AM05	Q	6.47	-	-	-	-	-	-	-	-	-	-	-	-	-
AM06	Q	5.71	-	-	2	-	1	-	2	-	-	-	-	-	-
BE06	Q	5.01	-	-	-	-	-	1	1	-	-	-	-	-	-
BE07	Q	4.86	-	-	-	-	-	-	-	-	-	-	-	-	-
DE06	Q	5.36	-	2	-	-	-	-	-	-	-	-	-	-	-
SA03	Q	5.08	2	-	-	-	-	-	-	-	-	-	-	-	-
SA08	Q	4.76	-	-	-	-	-	-	-	-	-	-	-	-	-
VA03	Q	3.91	-	-	-	-	-	-	-	-	-	-	-	-	-
VA07	Q	3.84	-	-	-	-	-	-	-	-	-	-	-	-	-

Supporting information table S3. Accumulated rainfall (mm) during the whole year (Rainfall annual), and only during the three previous months to the sampling campaigns (Rainfall 3-months).

Park	Date	Station	Rainfall annual	Rainfall 3-months
Ordesa	2013	Bielsa	1,734	355
		Torla	1,449	85
		Mean	1,592	220
	2014	Bielsa	1,362	324
		Torla	1,304	297
		Mean	1,333	311
Picos	2013	Soto de Sajambre	418	112
		Sotres	652	18
		Mean	535	65
	2014	Soto de Sajambre	1,109	220
		Sotres	583	25
		Mean	846	122

Supporting information table S4. Mean abiotic parameters for sampled plots in Parks and forests. Data from fall 2013, spring 2014, and total mean (Mean) of the two periods pooled. Water – soil water content in %, Temp – soil temperature in ºC, pH – soil pH, Alt – altitude in m a.s.l, se – standard errors, n – number of measures.

Park	Forest	Date	Water (se)	Temp (se)	pH (se)	Alt	n
Ordesa	Beech	2013	51.03 (2.42)	10.08 (0.59)	6.75 (0.17)		22
		2014	57.01 (2.17)	6.63 (0.27)	6.85 (0.14)		22
		Mean	54.19 (1.67)	8.36 (0.42)	6.80 (0.11)	1422.73 (20.27)	44
	Pine	2013	49.76 (2.91)	9.90 (0.71)	6.08 (0.16)		24
		2014	49.81 (2.19)	9.01 (0.50)	6.01 (0.17)		24
		Mean	49.79 (1.83)	9.46 (0.44)	6.05 (0.12)	1252.58 (28.49)	48
Picos	Beech	2013	40.63 (2.94)	10.08 (0.55)	5.92 (0.20)		24
		2014	42.66 (2.00)	12.26 (0.34)	5.65 (0.28)		24
		Mean	41.64 (1.77)	11.17 (0.36)	5.79 (0.17)	1087.83 (32.29)	48
	Oak	2013	26.68 (3.78)	11.74 (0.81)	5.42 (0.32)		9
		2014	36.83 (2.17)	13.72 (0.52)	4.58 (0.27)		9
		Mean	31.75 (2.45)	12.73 (0.53)	5.00 (0.23)	863.56 (74.16)	18
	Mixed	2013	36.07 (3.27)	11.97 (0.59)	7.02 (0.24)		14
		2014	37.59 (2.71)	15.08 (0.56)	6.60 (0.22)		14
		Mean	36.83 (2.09)	13.53 (0.50)	6.81 (0.16)	559.79 (88.13)	28
